# Hydroxybenzoic Acid Derivatives as Dual-Target Ligands: Mitochondriotropic Antioxidants and Cholinesterase Inhibitors

**DOI:** 10.3389/fchem.2018.00126

**Published:** 2018-04-23

**Authors:** Catarina Oliveira, Fernando Cagide, José Teixeira, Ricardo Amorim, Lisa Sequeira, Francesco Mesiti, Tiago Silva, Jorge Garrido, Fernando Remião, Santiago Vilar, Eugenio Uriarte, Paulo J. Oliveira, Fernanda Borges

**Affiliations:** ^1^CIQUP, Department of Chemistry and Biochemistry, Faculty of Sciences, University of Porto, Porto, Portugal; ^2^CNC, Center for Neuroscience and Cell Biology, UC-Biotech, University of Coimbra, Cantanhede, Portugal; ^3^Department of “Scienze della Salute”, University “Magna Græcia” of Catanzaro, Catanzaro, Italy; ^4^Department of Chemical Engineering, School of Engineering (ISEP), Polytechnic of Porto, Porto, Portugal; ^5^UCIBIO-REQUIMTE, Laboratory of Toxicology, Department of Biological Sciences, Faculty of Pharmacy, University of Porto, Porto, Portugal; ^6^Departamento de Química Orgánica, Facultad de Farmacia, Universidade de Santiago de Compostela, Santiago de Compostela, Spain; ^7^Instituto de Ciencias Químicas Aplicadas, Facultad de Ingeniería, Universidad Autónoma de Chile, Santiago, Chile

**Keywords:** hydroxybenzoic acids, oxidative stress, mitochondria-targeted antioxidants, cholinesterase inhibitors, acetyl and butyrylcholinesterase

## Abstract

Alzheimer's disease (AD) is a multifactorial age-related disease associated with oxidative stress (OS) and impaired cholinergic transmission. Accordingly, targeting mitochondrial OS and restoring cholinergic transmission can be an effective therapeutic strategy toward AD. Herein, we report for the first time dual-target hydroxybenzoic acid (HBAc) derivatives acting as mitochondriotropic antioxidants and cholinesterase (ChE) inhibitors. The studies were performed with two mitochondriotropic antioxidants **AntiOxBEN**_**1**_ (catechol derivative), and **AntiOxBEN**_**2**_ (pyrogallol derivative) and compounds **15–18**, which have longer spacers. Compounds **AntiOxBEN**_**1**_ and **15**, with a shorter carbon chain spacer (six- and eight-carbon) were shown to be potent antioxidants and BChE inhibitors (IC_50_ = 85 ± 5 and 106 ± 5 nM, respectively), while compounds **17** and **18** with a 10-carbon chain were more effective AChE inhibitors (IC_50_ = 7.7 ± 0.4 and 7.2 ± 0.5 μM, respectively). Interestingly, molecular modeling data pointed toward bifunctional ChEs inhibitors. The most promising ChE inhibitors acted by a non-competitive mechanism. In general, with exception of compounds **15** and **17**, no cytotoxic effects were observed in differentiated human neuroblastoma (SH-SY5Y) and human hepatocarcinoma (HepG2) cells, while Aβ-induced cytotoxicity was significantly prevented by the new dual-target HBAc derivatives. Overall, due to its BChE selectivity, favorable toxicological profile, neuroprotective activity and drug-like properties, which suggested blood-brain barrier (BBB) permeability, the mitochondriotropic antioxidant **AntiOxBEN**_**1**_ is considered a valid lead candidate for the development of dual acting drugs for AD and other mitochondrial OS-related diseases.

## Introduction

Alzheimer's disease (AD) is a multifactorial age-related disease, closely associated with impaired cholinergic transmission and oxidative stress (OS), among other factors (Guo et al., 2013; Zheng et al., 2014; Talevi, 2015; Nikolic et al., 2016). According to the cholinergic hypothesis, impairment in the cholinergic function is of critical importance in AD, especially in brain areas like the neocortex and the hippocampus, which control learning, memory, behavior and emotional responses. The levels of acetylcholine (ACh) in the synaptic cleft are tightly regulated by cholinesterases enzymes (ChEs): acetylcholinesterase (AChE) and butyrylcholinesterase (BChE) are the key regulators of cholinergic tone and transmission (Anand and Singh, 2013; Colović et al., 2013). While AChE in the healthy brain predominates, BChE is considered to play a minor role in the regulation of synaptic ACh levels (Li et al., 2017; Shah et al., 2017). However, in advanced stages of AD, AChE activity may decrease approximately by 50% in distinctive regions of the brain while BChE activity is enhanced, making both ChEs stimulating targets for the treatment of AD (Li et al., 2017; Shah et al., 2017). Moreover, it was proposed that low-activity BChE in AD patients correlates with better cognitive function (Holmes et al., 2005). Additionally, it has been shown that ChEs are proteins that colocalize with Aβ deposits and directly promotes Aβ assembly and aggregation into insoluble plaques, a classic biochemical hallmark of AD pathology (Morán et al., 1993). These secondary non-cholinergic functions of ChEs are attributed to the peripheral active site (PAS) of the enzyme's active site (Bajda et al., 2013; Silva et al., 2014). While deposition of Aβ plaques is the hallmark of the disease, the neurotoxicity of Aβ oligomers was shown to be stronger than that of the fibrils.

Brain is highly vulnerable to OS due to its high-energy demand and oxygen exposure, rich abundance of easily oxidizable polyunsaturated fatty acids, high level of potent reactive oxygen species (ROS), and relative paucity of endogenous antioxidants. OS and mitochondrial damage have been implicated in the pathogenesis of several age-related diseases (Guo et al., 2013). The redox alterations promoted by OS in specific cellular components lead to a more oxidized state, often resulting from an increased production of ROS and/or more limited intrinsic antioxidant activity (Dai et al., 2014; Dhawan, 2014; Bhat et al., 2015; Ksiazek-Winiarek and Głabinski, 2015). Therefore, it is believed that antioxidant therapy can operate as a pharmacological approach to prevent or delay the OS events that lead to neurodegeneration.

The fact that several drugs exert their effect through the interaction with diverse targets is shifting the drug discovery paradigm from the one target to a multiple-target approach. This approach is becoming increasingly important in drug discovery for multifactorial diseases, such as AD. As such, looking for new chemical entities that can minimize OS and restore cholinergic transmission by targeting ChEs can be a valid pharmacological strategy for the clinical management of AD.

**Figure 1:**
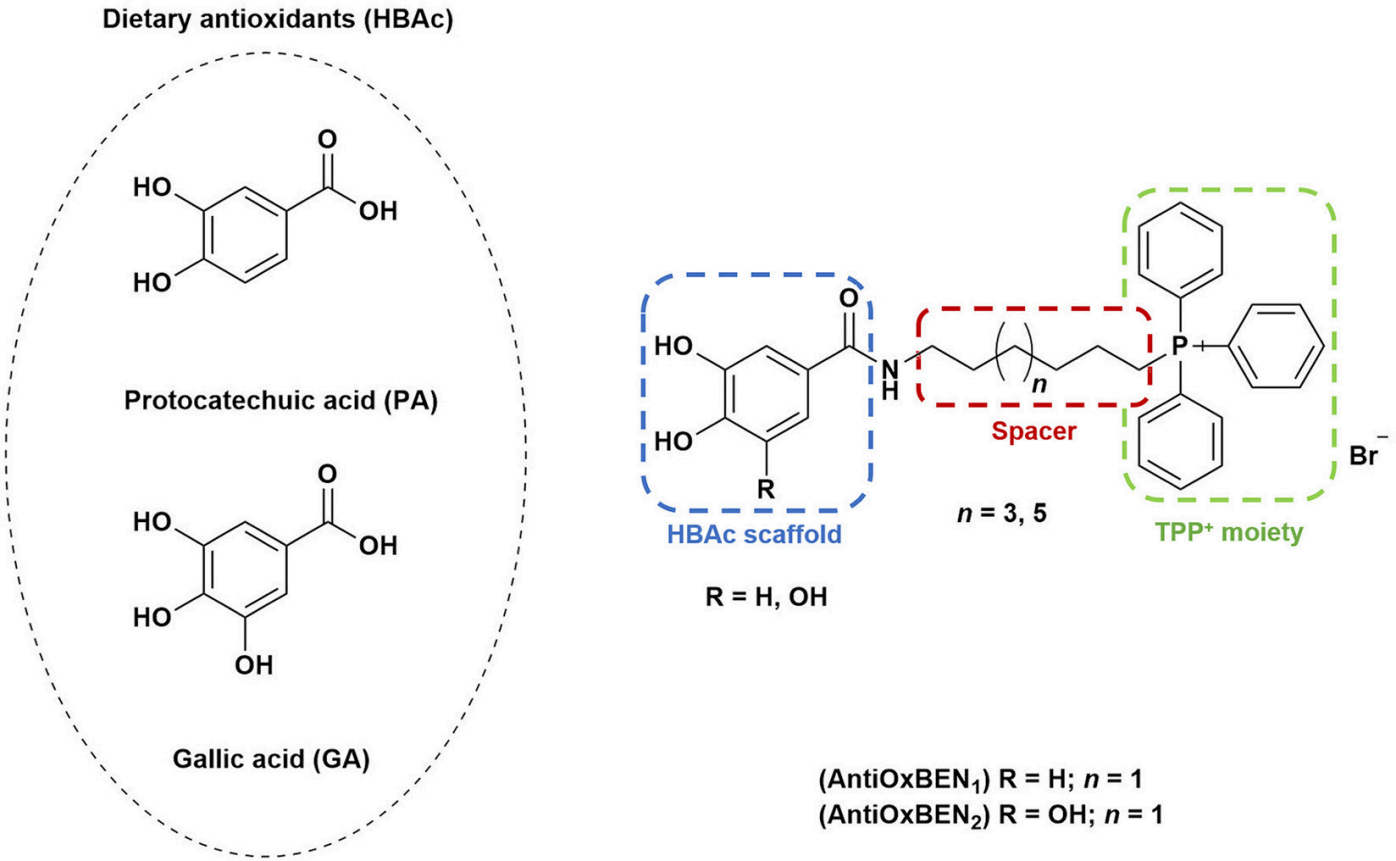
Rational design followed to develop novel dual target agents (ChE inhibitors and mitochondriotropic antioxidants).

Protocatechuic (PA, Figure 1) and gallic acids (GA, Figure 1) are hydroxybenzoic acids (HBAc) widely distributed in plants and fruit with diverse biological properties such as antioxidant, anti-inflammatory, antimicrobial and anticancer activity, as well as neuroprotection (Heleno et al., 2015; Pezzini et al., 2017; Szwajgier et al., 2017). Although this type of dietary antioxidants has promising *in vitro* outcomes, the translation in antioxidant therapy have had a dissatisfying clinical outcome, which has been directly associated with poor bioavailability, particularly inefficient cellular uptake and target selectivity (Guzman-Villanueva et al., 2015). To address this limitation, targeting mitochondria with organelle-specific molecules can be a useful therapeutic strategy for the prevention and/or treatment of OS-related diseases such as AD.

The design and synthesis of two mitochondriotropic antioxidants based on HBAc (**AntiOxBEN**_**1**_ and **AntiOxBEN**_**2**_, Figure 1), in which PA and GA were covalently bound to a triphenylphosphonium cation (TPP^+^) through a six-carbon aliphatic chain has been previously reported (Teixeira et al., 2017b). AntiOxBENs effectively accumulated in rat liver mitochondria, driven by the negative-inside organelle transmembrane electric potential (ΔΨ), and prevented lipid peroxidation while exhibiting low toxicity (Teixeira et al., 2017b). AntiOxBENs presented higher lipophilicity than the parent compounds (PA and GA), and similar antioxidant and iron chelating properties.

As part of our drug discovery program, and following an AD multi-target strategy, **AntiOxBEN**_**1**_ and **AntiOxBEN**_**2**_ were screened in this work toward ChEs. To perform structure-activity relationship studies the series was extended (Figure 1) and the antioxidant profile in cell free and cell-based systems as well as the inhibitory activities toward AChE and BChE of the new derivatives were evaluated. The cytotoxicity profile, drug-like properties and mechanism of enzymatic inhibition were also assessed. Moreover, to understand the enzyme(s)-inhibitor(s) interactions, molecular modeling studies were performed using models based in the crystal structures of the targets.

## Materials and methods

### Chemistry

#### Reagents and general conditions

All reagents were purchased from Sigma-Aldrich and TCI Chemicals. All solvents were *pro analysis* grade from Merck, Carlo Erba Reagents and Scharlab.

Thin layer chromatography (TLC) was performed on precoated silica gel 60 F254 acquired from Merck with layer thickness of 0.2 mm. Reaction control was monitored using ethyl acetate and/or ethyl acetate:methanol (9:1) and spots were visualized under UV detection at 254 and 366 nm. Following the extraction step, the organic layers were dried over anhydrous sodium sulfate. Flash column chromatography was carried out with silica gel 60 0.040–0.063 mm acquired from Carlo-Erba Reactifs. Cellulose flash column chromatography was carried out with cellulose powder 0.01–0.10 mm acquired from Sigma-Aldrich. The elution systems used for flash chromatography were specified for each compound. Solvents were evaporated using a Büchi Rotavapor.

#### Apparatus

NMR data were acquired on a Bruker Avance III 400 NMR spectrometer, at room temperature, operating at 400.15 MHz for ^1^H and 100.62 MHz for ^13^C and DEPT135 (Distortionless Enhancement by Polarization Transfer). Tetramethylsilane (TMS) was used as internal reference; chemical shifts (δ) were expressed in ppm and coupling constants (*J*) were given in Hz. DEPT135 values were included in ^13^C NMR data (underline values).

Mass spectra (MS) were carried out on a Varian 320-MS (EI) or Bruker Microtof (ESI) apparatus; the data were reported as *m/z* (% of relative intensity of the most important fragments).

#### Synthesis of benzoic based derivatives

##### General procedure used to obtain benzoic acid amide derivatives (3–6).

The appropriate benzoic acid (3,4-dimethoxybenzoic acid (**1**) or 3,4,5-trimethoxybenzoic acid (**2**), 1 mmol) was dissolved in dichloromethane (15 mL) and POCl_3_ (1 mmol) was added at room temperature. After 30 min, the reactional mixture was cooled (ice bath) and 8-aminooctan-1-ol or 10-aminodecan-1-ol (1.2 mmol) and DIPEA (4 mmol) were added. The reaction was stirred for 1–2 h at room temperature. The mixture was extracted with dichloromethane (3 × 20 mL). The organic phases were combined, washed with water, NaHCO_3_ 5% (20 mL) and HCl 1 M (20 mL). The organic phases were combined, dried and, after filtration, the solvent was evaporated and the compound purified by silica gel flash chromatography using ethyl acetate as eluting system. The fractions containing the intended compound were collected and the solvent was evaporated to dryness. The reaction was followed by TLC (silica gel, ethyl acetate). The procedure was adapted from the literature (Chen et al., 2013).

***N*-(8-Hydroxyoctyl)-3,4-dimethoxybenzamide (3)**. η = 49%. ^1^H RMN (CDCl_3_): δ = 1.29–1.38 (8H, *m*, N(CH_2_)_2_(CH_2_)_4_), 1.51–1.61 (4H, *m*, NCH_2_CH_2_(CH_2_)_4_CH_2_), 1.63 (1H, *s*, OH), 3.37–3.47 (2H, *m*, NCH_2_), 3.62 (2H, *t, J* = 6.6 Hz, CH_2_O), 3.91 (6H, *s*, 2 × OCH_3_), 6.19 (1H, *s*, NH), 6.84 (1H, *d, J* = 8.4 Hz, H(5)), 7.25 (1H, *dd, J* = 2.0, 8.4 Hz, H(6)), 7.41 (1H, *d, J* = 2.0 Hz, H(2)). ^13^C RMN (CDCl_3_): δ = 25.8 (N(CH_2_)_5_CH_2_), 27.0 (N(CH_2_)_2_CH_2_), 29.3 (N(CH_2_)_3_CH_2_), 29.4 (N(CH_2_)_4_CH_2_), 29.8 (NCH_2_CH_2_), 32.8 (N(CH_2_)_6_CH_2_), 40.2 (NCH_2_), 56.1 (2 × OCH_3_), 63.0 (CH_2_O), 110.4 (C(5)), 110.8 (C(2)), 119.2 (C(6)), 127.6 (C(1)), 149.1 (C(3)), 151.7 (C(4)), 167.2 (CO). ESI/MS *m/z* (%): 332 ([M+Na]^+^, 100), 310 ([M+H]^+^, 67), 165 (97).

***N*-(8-Hydroxyoctyl)-3,4,5-trimethoxybenzamide (4)**. η = 71%. ^1^H RMN (CDCl_3_): δ = 1.28–1.42 (8H, *m*, N(CH_2_)_2_(CH_2_)_4_), 1.49–1.66 (4H, *m*, NCH_2_CH_2_(CH_2_)_4_CH_2_), 1.73 (1H, *s*, OH), 3.37–3.49 (2H, *m*, NCH_2_), 3.62 (2H, *t, J* = 6.6 Hz, CH_2_O), 3.87 (3H, *s*, OCH_3_), 3.89 (6H, *s*, 2 × OCH_3_), 6.16 (1H, *s*, NH), 6.98 (2H, *s*, H(2) and H(6)). ^13^C RMN (CDCl_3_): δ = 25.8 (N(CH_2_)_5_CH_2_), 27.0 (N(CH_2_)_2_CH_2_), 29.3 (N(CH_2_)_3_CH_2_), 29.4 (N(CH_2_)_4_CH_2_), 29.8 (NCH_2_CH_2_), 32.8 (N(CH_2_)_6_CH_2_), 40.3 (NCH_2_), 56.5 (2 × OCH_3_), 61.0 (OCH_3_), 63.1 (CH_2_O), 104.5 (C(2) and C(6)), 130.5 (C(1)), 141.0 (C(4)), 153.3 (C(3) and C(5)), 167.4 (CO). ESI/MS *m/z* (%): 362 ([M+Na]^+^, 57), 340 ([M+H]^+^, 98), 195 (100), 154 (54).

***N*-(10-Hydroxydecyl)-3,4-dimethoxybenzamide (5)**. η = 54%. ^1^H RMN (CDCl_3_): δ = 1.24–1.35 (12H, *m*, N(CH_2_)_2_(CH_2_)_6_), 1.50–1.60 (4H, *m*, NCH_2_CH_2_(CH_2_)_6_CH_2_), 1.61 (1H, *s*, OH), 3.36–3.46 (2H, *m*, NCH_2_), 3.62 (2H, *t, J* = 6.6 Hz, CH_2_O), 3.90 (6H, *s*, 2 × OCH_3_), 6.17 (1H, *s*, NH), 6.84 (1H, *d, J* = 8.4 Hz, H(5)), 7.25 (1H, *dd, J* = 2.0, 8.4 Hz, H(6)), 7.41 (1H, *d, J* = 2.0 Hz, H(2)). ^13^C RMN (CDCl_3_): δ = 25.8 (N(CH_2_)_7_CH_2_), 27.1 (N(CH_2_)_2_CH_2_), 29.4 (N(CH_2_)_3_CH_2_), 29.5 (N(CH_2_)_4_CH_2_CH_2_CH_2_), 29.6 (N(CH_2_)_5_CH_2_), 29.8 (NCH_2_CH_2_), 32.9 (N(CH_2_)_8_CH_2_), 40.2 (NCH_2_), 56.1 (2 × OCH_3_), 63.1 (CH_2_O), 110.4 (C(5)), 110.8 (C(2)), 119.2 (C(6)), 127.7 (C(1)), 149.1 (C(3)), 151.7 (C(4)), 167.2 (CO). ESI/MS *m/z* (%): 360 ([M+Na]^+^, 43), 338 ([M+H]^+^, 61), 165 (100).

***N*-(10-Hydroxydecyl)-3,4,5-trimethoxybenzamide (6)**. η = 71%. ^1^H RMN (CDCl_3_): δ = 1.23–1.36 (12H, *m*, N(CH_2_)_2_(CH_2_)_6_), 1.49–1.62 (4H, *m*, NCH_2_CH_2_(CH_2_)_6_CH_2_), 1.64 (1H, *s*, OH), 3.36–3.49 (2H, *m*, NCH_2_), 3.63 (2H, *t, J* = 6.6 Hz, CH_2_O), 3.86 (3H, *s*, OCH_3_), 3.89 (6H, *s*, 2 × OCH_3_), 6.11 (1H, *s*, NH), 6.98 (2H, *s*, H(2) and H(6)). ^13^C RMN (CDCl_3_): δ = 25.8 (N(CH_2_)_7_CH_2_), 27.1 (N(CH_2_)_2_CH_2_), 29.4 (N(CH_2_)_3_CH_2_), 29.5 (N(CH_2_)_4_CH_2_CH_2_CH_2_), 29.6 (N(CH_2_)_5_CH_2_), 29.8 (NCH_2_CH_2_), 32.9 (N(CH_2_)_8_CH_2_), 40.4 (NCH_2_), 56.5 (2 × OCH_3_), 61.0 (OCH_3_), 63.1 (CH_2_O), 104.5 (C(2) and C(6)), 130.5 (C(1)), 141.0 (C(4)), 153.3 (C(3) and C(5)), 167.4 (CO). ESI/MS *m/z* (%): 368 ([M+H]^+^, 100), 195 (98), 169 (22), 154 (51).

##### General procedure used to obtain bromo derivatives (7–10)

Benzoic acid amide derivative (**3–6**) (1 mmol) and 1,2-dibromotetrachloroethane (1 mmol) were dissolved in THF (20 mL). After, 1,2-bis(diphenylphosphine)ethane (diphos) (0.5 mmol) was added and the reaction was stirred at room temperature for 20 h. Then, the reaction mixture was filtered using a Celite pad. After evaporation of the filtrate, an oil residue was obtained. The oil was purified by silica gel flash chromatography using ethyl acetate as eluting system. The fractions containing the intended compound were collected. After the solvent evaporation, the compound was recrystallized from *n*-hexane. The reaction was followed by TLC (silica gel, ethyl acetate). The procedure was adapted from the literature (Pollastri et al., 2001; Teixeira et al., 2017b).

***N*-(8-Bromooctyl)-3,4-dimethoxybenzamide (7)**. η = 64%. ^1^H RMN (CDCl_3_): δ = 1.30–1.45 (8H, *m*, N(CH_2_)_2_(CH_2_)_4_), 1.55–1.65 (2H, *m*, NCH_2_CH_2_), 1.79–1.89 (2H, *m*, N(CH_2_)_6_CH_2_), 3.39 (2H, *t, J* = 6.8 Hz, CH_2_Br), 3.40–3.45 (2H, *m*, NCH_2_), 3.91 (6H, *s*, 2 × OCH_3_), 6.12 (1H, *s*, Hz, NH), 6.84 (1H, *d, J* = 8.4 Hz, H(5)), 7.25 (1H, *dd, J* = 2.0, 8.4 Hz, H(6)), 7.42 (1H, *d, J* = 2.0 Hz, H(2)). ^13^C RMN (CDCl_3_): δ = 27.0 (N(CH_2_)_2_CH_2_), 28.2 (N(CH_2_)_5_CH_2_), 28.8 (N(CH_2_)_4_CH_2_), 29.2 (N(CH_2_)_3_CH_2_), 29.8 (NCH_2_CH_2_), 32.9 (N(CH_2_)_6_CH_2_), 34.1 (CH_2_Br), 40.2 (NCH_2_), 56.1 (2 × OCH_3_), 110.4 (C(5)), 110.8 (C(2)), 119.2 (C(6)), 127.7 (C(1)), 149.2 (C(3)), 151.8 (C(4)), 167.2 (CO). ESI/MS *m/z* (%): 396 ([M+Na+2]^+^, 30), 394 ([M+Na]^+^, 30), 374 ([M+H+2]^+^, 100), 372 ([M+H]^+^, 91), 165 (32), 139 (27), 124 (40).

***N*-(8-Bromooctyl)-3,4,5-trimethoxybenzamide (8)**. η = 69%. ^1^H RMN (CDCl_3_): δ = 1.27–1.47 (8H, *m*, N(CH_2_)_2_(CH_2_)_4_), 1.56–1.65 (2H, *m*, NCH_2_CH_2_), 1.79–1.90 (2H, *m*, N(CH_2_)_6_CH_2_), 3.40 (2H, *t, J* = 6.8 Hz, CH_2_Br), 3.41–3.47 (2H, *m*, NCH_2_), 3.87 (3H, *s*, OCH_3_), 3.89 (6H, *s*, 2 × OCH_3_), 6.12 (1H, *s*, NH), 6.98 (2H, *s*, H(2) and H(6)). ^13^C RMN (CDCl_3_): δ = 27.0 (N(CH_2_)_2_CH_2_), 28.2 (N(CH_2_)_5_CH_2_), 28.8 (N(CH_2_)_4_CH_2_), 29.2 (N(CH_2_)_3_CH_2_), 29.8 (NCH_2_CH_2_), 32.8 (N(CH_2_)_6_CH_2_), 34.1 (CH_2_Br), 40.3 (NCH_2_), 56.5 (2 × OCH_3_), 61.0 (OCH_3_), 104.5 (C(2) and C(6)), 130.4 (C(1)), 141.0 (C(4)), 153.3 (C(3) and C(5)), 167.4 (CO). ESI/MS *m/z* (%): 426 ([M+Na+2]^+^, 35), 424 ([M+Na]^+^, 39), 404 ([M+H+2]^+^, 78), 402 ([M+H]^+^, 93), 195 (26), 169 (25), 154 (100).

***N*-(10-Bromodecyl)-3,4-dimethoxybenzamide (9)**. η = 62%. ^1^H RMN (CDCl_3_): δ = 1.24–1.44 (12H, *m*, N(CH_2_)_2_(CH_2_)_6_), 1.55–1.66 (2H, *m*, NCH_2_CH_2_), 1.78–1.88 (2H, *m*, N(CH_2_)_8_CH_2_), 3.39 (2H, *t, J* = 6.9 Hz, CH_2_Br), 3.41–3.46 (2H, *m*, NCH_2_), 3.90 (6H, *s*, 2 × OCH_3_), 6.83 (1H, *d, J* = 8.4 Hz, H(5)), 7.30 (1H, *dd, J* = 2.0, 8.4 Hz, H(6)), 7.42 (1H, *d, J* = 2.0 Hz, H(2)). ^13^C RMN (CDCl_3_): δ = 27.1 (N(CH_2_)_2_CH_2_), 28.2 (N(CH_2_)_7_CH_2_), 28.8 (N(CH_2_)_6_CH_2_), 29.4 (N(CH_2_)_3_CH_2_), 29.5 (N(CH_2_)_4_(CH_2_)_2_), 29.7 (NCH_2_CH_2_), 32.9 (N(CH_2_)_8_CH_2_), 34.1 (CH_2_Br), 40.6 (NCH_2_), 56.2 (2 × OCH_3_), 110.4 (C(5)), 110.9 (C(2)), 119.9 (C(6)), 126.3 (C(1)), 149.1 (C(3)), 152.2 (C(4)), 167.9 (CO). ESI/MS *m/z* (%): 424 ([M+Na+2]^+^, 20), 422 ([M+Na]^+^, 23), 402 ([M+H+2]^+^, 100), 400 ([M+H]^+^, 99), 165 (20), 139 (21), 124 (22).

***N*-(10-Bromodecyl)-3,4,5-trimethoxybenzamide (10)**. η = 63%. ^1^H RMN (CDCl_3_): δ = 1.26–1.46 (12H, *m*, N(CH_2_)_2_(CH_2_)_6_), 1.55–1.68 (2H, *m*, NCH_2_CH_2_), 1.77–1.90 (2H, *m*, N(CH_2_)_8_CH_2_), 3.40 (2H, *t, J* = 6.9 Hz, CH_2_Br), 3.40–3.48 (2H, *m*, NCH_2_), 3.87 (3H, *s*, OCH_3_), 3.90 (6H, *s*, 2 × OCH_3_), 6.13 (1H, *s*, NH), 6.98 (2H, *s*, H(2) and H(6)). ^13^C RMN (CDCl_3_): δ = 27.1 (N(CH_2_)_2_CH_2_), 28.3 (N(CH_2_)_7_CH_2_), 28.8 (N(CH_2_)_6_CH_2_), 29.4 (N(CH_2_)_3_CH_2_), 29.4 (N(CH_2_)_4_(CH_2_)_2_), 29.8 (NCH_2_CH_2_), 32.9 (N(CH_2_)_8_CH_2_), *34.1* (CH_2_Br), 40.4 (NCH_2_), 56.5 (2 × OCH_3_), 61.0 (OCH_3_), 104.5 (C(2) and C(6)), 130.4 (C(1)), 141.0 (C(4)), 153.3 (C(3) and C(5)), 167.4 (CO). ESI/MS *m/z* (%): 432 ([M+H+2]^+^, 17), 430 ([M+H]^+^, 13), 195 (23), 169 (25), 154 (100).

##### General procedure used to obtain triphenylphosphonium salts (11–14)

The appropriate bromo derivative (**7–10**) (1 mmol) was mixed with triphenylphosphine (PPh_3_, 1 mmol) in a round-bottomed flask and heated to 100°C for 48 h. The residue was purified by silica gel flash chromatography using gradient elution (ethyl acetate:methanol from 9:1 to 6:4). The fractions containing the desired compound were collected and the solvent was evaporated to dryness. The reaction was followed by TLC [silica gel, ethyl acetate:methanol (9:1) and dichloromethane:methanol (9:1)]. The procedure was adapted from the literature (Brown et al., 2007; Teixeira et al., 2017b).

**(8-(3,4-Dimethoxybenzamido)octyl)triphenylphosphonium bromide (11)**. η = 84%. ^1^H NMR (CDCl_3_): δ = 1.23–1.43 (6H, *m*, NCH_2_(CH_2_)_3_), 1.57–1.71 (6H, *m*, NCH_2_CH_2_(CH_2_)_3_(CH_2_)_2_), 3.39–3.46 (2H, *m*, NCH_2_), 3.68–3.78 (2H, *m*, CH_2_P^+^), 3.87 (3H, *s*, OCH_3_), 3.95 (3H, *s*, OCH_3_), 6.87 (1H, *d, J* = 8.3 Hz, H(5)), 7.53 (1H, *s*, CO), 7.60 – 7.87 (17H, *m*, H(2), H(6) and PPh_3_). ^13^C NMR (CDCl_3_): δ = 22.5 (*d, J*_CP_ = 4.7 Hz, CH_2_(CH_2_)_2_P^+^), 22.8 (*d, J*_CP_ = 49.4 Hz, CH_2_P^+^), 26.2 (N(CH_2_)_2_CH_2_), 28.0 (N(CH_2_)_4_CH_2_), 28.2 (N(CH_2_)_3_CH_2_), 29.0 (NCH_2_CH_2_), 29.8 (*d, J*_CP_ = 15.9 Hz, *C*H_2_CH_2_P^+^), 39.9 (NCH_2_), 56.1 (OCH_3_), 56.6 (OCH_3_), 110.5 (C(5)), 111.1 (C(2)), 118.6 (*d, J*_CP_ = 85.8 Hz, 3 × C(1′)), 120.7 (C(6)), 127.6 (C(1)), 130.6 (*d, J*_CP_ = 12.5 Hz, 3 × C(3′) and 3 × C(5′)), 133.8 (*d, J*_CP_ = 10.0 Hz, 3 × C(2′) and 3 × C(6′)), 135.1 (*d, J*_CP_ = 3.0 Hz, 3 × C(4′)), 148.8 (C(3)), 151.4 (C(4)), 167.2 (CO). ESI/MS *m/z* (%): 555 ([M+H-Br]^+^, 52), 554 ([M-Br]^+^, 100).

**(8-(3,4,5-Trimethoxybenzamido)octyl)triphenylphosphonium bromide (12)**. η = 87%. ^1^H NMR (CDCl_3_): δ = 1.29–1.45 (6H, *m*, NCH_2_(C*H*_2_)_3_), 1.60–1.74 (6H, *m*, NCH_2_CH_2_(CH_2_)_3_(CH_2_)_2_), 3.43–3.50 (2H, *m*, NCH_2_), 3.65–3.74 (2H, *m*, CH_2_P^+^), 3.85 (3H, *s*, OCH_3_), 3.95 (6H, *s*, 2 × OCH_3_), 7.39 (2H, *s*, H(2) and H(6)), 7.66–7.83 (15H, *m*, PPh_3_), 8.12 (1H, *s*, NH). ^13^C NMR (CDCl_3_): δ = 22.4 (*d, J*_CP_ = 4.6 Hz, CH_2_(CH_2_)_2_P^+^), 22.7 (*d, J*_CP_ = 49.2 Hz, CH_2_P^+^), 25.9 (N(CH_2_)_2_CH_2_), 27.6 (N(CH_2_)_4_CH_2_), 27.9 (N(CH_2_)_3_CH_2_), 28.7 (NCH_2_CH_2_), 29.6 (*d, J*_CP_ = 16.1 Hz, CH_2_CH_2_P^+^), 40.0 (NCH_2_), 57.0 (2 × OCH_3_), 60.9 (OCH_3_), 105.3 (C(2) and C(6)), 118.5 (*d, J*_CP_ = 85.9 Hz, 3 × C(1′)), 130.5 (C(1)), 130.6 (*d, J*_CP_ = 12.5 Hz, 3 × C(3′) and 3 × C(5′)), 133.8 (*d, J*_CP_ = 10.0 Hz, 3 × C(2′) and 3 × C(6′)), 135.2 (*d, J*_CP_ = 3.0 Hz, 3 × C(4′)), 140.4 (C(4)), 153.0 (C(3) and C(5)), 167.2 (CO). ESI/MS *m/z* (%): 585 ([M+H-Br]^+^, 48), 584 ([M-Br]^+^, 100).

**(10-(3,4-Dimethoxybenzamido)decyl)triphenylphosphonium bromide (13)**. η = 82 %. ^1^H NMR (CDCl_3_): δ = 1.17 – 1.37 (12H, *m*, NCH_2_(CH_2_)_6_), 1.57 – 1.67 (4H, *m*, NCH_2_CH_2_(CH_2_)_6_CH_2_), 3.37 – 3.46 (2H, *m*, NCH_2_), 3.71 – 3.83 (2H, *m*, CH_2_P^+^), 3.89 (3H, *s*, OCH_3_), 3.93 (3H, *s*, OCH_3_), 6.86 (1H, *d, J* = 8.4 Hz, H(5)), 7.00 (1H, *s*, NH), 7.48 (1H, *d, J* = 8.4 Hz, H(6)), 7.55 (1H, *d, J* = 1.8 Hz, H(2)), 7.66–7.87 (15H, *m*, PPh_3_). ^13^C NMR (CDCl_3_): δ = 22.7 (*d, J*_CP_ = 4.6 Hz, CH_2_(CH_2_)_2_P^+^), 22.9 (*d, J*_CP_ = 49.6 Hz, CH_2_P^+^), 26.7 (N(CH_2_)_2_CH_2_), 28.7 (N(CH_2_)_6_CH_2_), 28.8 (N(CH_2_)_3_(CH_2_)_3_), 29.4 (NCH_2_CH_2_), 30.2 (*d, J*_CP_ = 15.8 Hz, CH_2_CH_2_P^+^), 40.1 (NCH_2_), 56.1 (OCH_3_), 56.4 (OCH_3_), 110.5 (C(5)), 111.0 (C(2)), 118.6 (*d, J*_CP_ = 85.8 Hz, 3 × C(1′)), 120.2 (C(6)), 127.5 (C(1)), 130.6 (*d, J*_CP_ = 12.5 Hz, 3 × C(3′) and 3 × C(5′)), 133.8 (*d, J*_CP_ = 9.9 Hz, 3 × C(2′) and 3 × C(6′)), 135.1 (*d, J*_CP_ = 2.9 Hz, 3 × C(4′)), 148.9 (C(3)), 151.5 (C(4)), 167.2 (CO). ESI/MS *m/z* (%): 583 ([M+H-Br]^+^, 38), 582 ([M-Br]^+^, 100).

**(10-(3,4,5-Trimethoxybenzamido)decyl)triphenylphosphonium bromide (14)**. η = 92%. ^1^H NMR (CDCl_3_): δ = 1.18–1.38 (12H, *m*, NCH_2_(CH_2_)_6_), 1.56–1.64 (4H, *m*, NCH_2_CH_2_(CH_2_)_6_CH_2_), 3.39–3.46 (2H, *m*, NCH_2_), 3.68–3.76 (2H, *m*, CH_2_P^+^), 3.84 (3H, *s*, OCH_3_), 3.90 (6H, *s*, 2 × OCH_3_), 7.27 (2H, *s*, H(2) and H(6)), 7.60 (1H, *s*, NH), 7.65–7.85 (15H, *m*, PPh_3_). ^13^C NMR (CDCl_3_): δ = 22.6 (*d, J*_CP_ = 4.3 Hz, CH_2_(CH_2_)_2_P^+^), 22.8 (*d, J*_CP_ = 49.9 Hz, CH_2_P^+^), 26.5 (N(CH_2_)_2_CH_2_), 28.2 (N(CH_2_)_6_CH_2_), 28.5 (N(CH_2_)_3_(CH_2_)_3_), 29.0 (NCH_2_CH_2_), 29.9 (*d, J*_CP_ = 15.8 Hz, CH_2_CH_2_P^+^), 40.1 (NCH_2_), 56.7 (2 × OCH_3_), 60.8 (OCH_3_), 105.0 (C(2) and C(6)), 118.4 (*d, J*_CP_ = 85.8 Hz, 3 × C(1′)), 130.3 (C(1)), 130.5 (*d, J*_CP_ = 12.5 Hz, 3 × C(3′) and 3 × C(5′)), 133.7 (*d, J*_CP_ = 9.9 Hz, 3 × C(2′) and 3 × C(6′)), 135.1 (*d, J*_CP_ = 3.0 Hz, 3 × C(4′)), 140.3 (C(4)), 152.9 (C(3) and C(5)), 167.1 (CO). ESI/MS *m/z* (%): 613 ([M+H-Br]^+^, 51), 612 ([M-Br]^+^, 100).

##### General procedure used to obtain mitochondriotropic antioxidants (15–18).

Triphenylphosphonium salt derivative (**11–14**) (1 mmol) was dissolved in anhydrous dichloromethane (15 mL). The reaction mixture was stirred under argon and cooled at a temperature below −70°C. To this solution, boron tribromide (5–7 mmol, 1 M solution in dichloromethane) was added and the reaction was kept at −70°C for 10 min. After reaching room temperature, the reaction was continued for 12 h. Thereafter, the reaction was finished by cautious addition of water (40 mL). After removing the water, the product was dissolved in methanol and the solvent evaporated. The residue was purified by cellulose flash chromatography using gradient elution (dichloromethane:methanol from 9:1 to 6:4). The fractions containing the desired compound were collected and the solvent was evaporated to dryness. The reaction was followed by TLC [silica gel, mobile phase with dichloromethane:methanol (9:1)]. The procedure was adapted from the literature (Milhazes et al., 2006; Teixeira et al., 2012, 2017a).

**(8-(3,4-Dihydroxybenzamido)octyl)triphenylphosphonium bromide (15)**. η = 82 %. ^1^H NMR (DMSO-*d*_6_): δ = 1.13 – 1.35 (6H, *m*, N(CH_2_)_2_(CH_2_)_3_), 1.36–1.72 (6H, *m*, NCH_2_CH_2_(CH_2_)_3_(CH_2_)_2_), 3.09–3.20 (2H, *m*, NCH_2_), 3.49–3.63 (2H, *m*, CH_2_P^+^), 6.74 (1H, *d, J* = 8.2 Hz, H(5)), 7.16 (1H, *dd, J* = 2.0, 8.2 Hz, H(6)), 7.26 (1H, *d, J* = 2.0 Hz, H(2)), 7.71–7.93 (15H, *m*, PPh_3_), 8.09 (1H, *s*, NH). ^13^C NMR (DMSO-*d*_6_): δ = 20.2 (*d, J*_CP_ = 49.9 Hz, CH_2_P^+^), 21.8 (*d, J*_CP_ = 4.4 Hz, CH_2_(CH_2_)_2_P^+^), 26.3 (N(CH_2_)_2_CH_2_), 28.0 (N(CH_2_)_4_CH_2_), 28.4 (N(CH_2_)_3_CH_2_), 29.2 (NCH_2_CH_2_), 29.7 (*d, J*_CP_ = 16.5 Hz, CH_2_CH_2_P^+^), 39.0 (NCH_2_), 114.8 (C(5)), 115.1 (C(2)), 118.6 (*d, J*_CP_ = 85.6 Hz, 3 × C(1′)), 118.8 (C(6)), 126.0 (C(1)), 130.2 (*d, J*_CP_ = 12.4 Hz, 3 × C(3′) and 3 × C(5′)), 133.6 (*d, J*_CP_ = 10.1 Hz, 3 × C(2′), and 3 × C(6′)), 134.9 (*d, J*_CP_ = 2.8 Hz, 3 × C(4′)), 144.7 (C(3)), 148.1 (C(4)), 166.0 (CO). ESI/MS *m/z* (%): 527 ([M+H-Br]^+^, 55), 526 ([M-Br]^+^, 100). ESI/HRMS calcd for C_33_H_37_NO_3_P^+^ (M^+^): 526.2506, found 526.2633.

**(8-(3,4,5-Trihydroxybenzamido)octyl)triphenylphosphonium bromide (16)**. η = 83%. ^1^H NMR (DMSO-*d*_6_): δ = 1.13 – 1.32 (6H, *m*, N(CH_2_)_2_(CH_2_)_3_), 1.36 – 1.60 (6H, *m*, NCH_2_CH_2_(CH_2_)_3_(CH_2_)_2_), 3.06–3.20 (2H, *m*, NCH_2_), 3.39–3.66 (5H, *m*, CH_2_P^+^ and 3 × OH), 6.80 (2H, *s*, H(2) and H(6)), 7.71–7.93 (15H, *m*, PPh_3_), 7.99 (1H, *s*, NH). ^13^C NMR (DMSO-*d*_6_): δ = 20.1 (*d, J*_CP_ = 49.3 Hz, CH_2_P^+^), 21.7 (*d, J*_CP_ = 4.2 Hz, CH_2_(CH_2_)_2_P^+^), 26.3 (N(CH_2_)_2_CH_2_), 28.0 (N(CH_2_)_4_CH_2_), 28.4 (N(CH_2_)_3_CH_2_), 29.1 (NCH_2_CH_2_), 29.7 (*d, J*_CP_ = 16.4 Hz, CH_2_CH_2_P^+^), 38.9 (NCH_2_), 106.7 (C(2) and C(6)), 118.6 (*d, J*_CP_ = 85.6 Hz, 3 × C(1′)), 125.2 (C(1)), 130.2 (*d, J*_CP_ = 12.4 Hz, 3 × C(3′) and 3 × C(5′)), 133.6 (*d, J*_CP_ = 10.1 Hz, 3 × C(2′) and 3 × C(6′)), 134.9 (*d, J*_CP_ = 2.8 Hz, 3 × C(4′)), 136.0 (C(4)), 145.3 (C(3) and C(5)), 166.2 (CO). ESI/MS *m/z* (%): 543 ([M+H-Br]^+^, 100), 542 ([M-Br]^+^, 59). ESI/HRMS calcd for C_33_H_37_NO_4_P^+^ (M^+^): 542.2455, found 542.2412.

**(10-(3,4-Dihydroxybenzamido)decyl)triphenylphosphonium bromide (17)**. η = 69%. ^1^H NMR (DMSO-*d*_6_): δ = 1.11–1.30 (10H, *m*, N(CH_2_)_2_(CH_2_)_5_), 1.36–1.61 (6H, *m*, NCH_2_CH_2_(CH_2_)_5_(CH_2_)_2_), 3.10–3.25 (2H, *m*, NCH_2_), 3.46–3.62 (4H, *m*, CH_2_P^+^ and 2 × OH), 6.73 (1H, *d, J* = 8.2 Hz, H(5)), 7.16 (1H, *dd, J* = 2.1, 8.2 Hz, H(6)), 7.25 (1H, *d, J* = 2.1 Hz, H(2)), 7.74–7.91 (15H, *m*, PPh_3_), 8.07 (1H, *t, J* = 5.6 Hz, NH). ^13^C NMR (DMSO-*d*_6_): δ = 20.1 (*d, J*_CP_ = 50.2 Hz, CH_2_P^+^), 21.7 (*d, J*_CP_ = 4.7 Hz, CH_2_(CH_2_)_2_P^+^), 26.5 (N(CH_2_)_2_CH_2_), 28.0 (N(CH_2_)_6_CH_2_), 28.6 (N(CH_2_)_3_CH_2_), 28.7 (N(CH_2_)_5_CH_2_), 28.8 (N(CH_2_)_4_CH_2_), 29.2 (NCH_2_CH_2_), 29.8 (*d, J*_CP_ = 16.4 Hz, CH_2_CH_2_P^+^), 39.0 (NCH_2_), 114.7 (C(5)), 115.0 (C(2)), 118.6 (*d, J*_CP_ = 85.6 Hz, 3 × C(1′)), 118.8 (C(6)), 126.0 (C(1)), 130.2 (*d, J*_CP_ = 12.4 Hz, 3 × C(3′) and 3 × C(5′)), 133.6 (*d, J*_CP_ = 10.1 Hz, 3 × C(2′) and 3 × C(6′)), 134.9 (*d, J*_CP_ = 3.0 Hz, 3 × C(4′)), 144.7 (C(3)), 148.1 (C(4)), 165.9 (CO). ESI/MS *m/z* (%): 555 ([M+H-Br]^+^, 58), 554 ([M-Br]^+^, 100). ESI/HRMS calcd for C_35_H_41_NO_3_P^+^ (M^+^): 554.2819, found 554.2821.

**(10-(3,4,5-Trihydroxybenzamido)decyl)triphenylphosphonium bromide (18)**. η = 89%. ^1^H NMR (DMSO-*d*_6_): δ = 1.02–1.30 (10H, *m*, N(CH_2_)_2_(CH_2_)_5_), 1.34–1.59 (6H, *m*, NCH_2_CH_2_(CH_2_)_5_(CH_2_)_2_), 3.06–3.20 (2H, *m*, NCH_2_), 3.46–3.64 (2H, *m*, CH_2_P^+^), 6.80 (2H, *s*, H(2) and H(6)), 7.69–7.93 (15H, *m*, PPh_3_), 8.00 (1H, *s*, NH). ^13^C NMR (DMSO-*d*_6_): δ = 20.2 (*d, J*_CP_ = 50.1 Hz, CH_2_P^+^), 21.7 (*d, J*_CP_ = 4.2 Hz, CH_2_(CH_2_)_2_P^+^), 26.4 (N(CH_2_)_2_CH_2_), 28.0 (N(CH_2_)_6_CH_2_), 28.6 (N(CH_2_)_3_CH_2_), 28.7 (N(CH_2_)_5_CH_2_), 28.8 (N(CH_2_)_4_CH_2_), 29.2 (NCH_2_CH_2_), 29.7 (*d, J*_CP_ = 16.4 Hz, CH_2_CH_2_P^+^), 39.0 (NCH_2_), 106.7 (C(2) and C(6)), 118.6 (*d, J*_CP_ = 85.6 Hz, 3 × C(1′)), 125.2 (C(1)), 130.2 (*d, J*_CP_ = 12.4 Hz, 3 × C(3′) and 3 × C(5′)), 133.6 (*d, J*_CP_ = 10.1 Hz, 3 × C(2′) and 3 × C(6′)), 134.9 (*d, J*_CP_ = 2.8 Hz, 3 × C(4′)), 136.0 (C(4)), 14537 (C(3) and C(5)), 166.2 (CO). ESI/MS *m/z* (%): 571 ([M+H-Br]^+^, 100), 570 ([M-Br]^+^, 13). ESI/HRMS calcd for C_35_H_41_NO_4_P^+^ (M^+^): 570.2768, found 570.2885.

#### Evaluation of radical scavenging activity

The radical scavenging activity of compounds **AntiOxBEN**_**1**_, **AntiOxBEN**_**2**_, and **15**–**18** was evaluated using the total antioxidant capacity spectrophotometric assays based on the DPPH^•^ (2,2′-diphenyl-1-picrylhydrazyl) and ABTS^•+^ (2,2′-azino-bis(3-ethylbenzthiazoline-6-sulfonic acid)) radicals.

##### DPPH^•^ radical assay

DPPH^•^ radical scavenging activity was evaluated as previously described (Teixeira et al., 2017b). The IC_50_ values were determined in triplicate from the dose-response inhibition curves and are expressed as mean ± standard deviation (SD).

##### ABTS^•+^ radical cation assay

ABTS^•+^ scavenging activity was evaluated as previously described (Teixeira et al., 2017b). The IC_50_ values were determined in triplicate from the dose-response inhibition curves and are expressed as mean ± standard deviation (SD).

#### Evaluation of redox properties

Voltammetric studies were performed using an Autolab PGSTAT12 potentiostat/galvanostat (Metrohm Autolab, Netherland) and a one-compartment glass electrochemical cell. Voltammetric curves were recorded at room temperature using a three-electrode system, according the conditions previously described (Teixeira et al., 2017b).

### Pharmacology

#### Reagents and general conditions

Acetylcholinesterase (AChE) from *Electrophorus electricus* (electric eel), butyrylcholinesterase (BChE) and acetylthiocholine iodide (ATCI), butyrylthiocholine iodide (BTCI), 5,5′-dithiobis-(2-nitrobenzoate) (DTNB), (4,5-dimethylthiazol-2-yl)-2,5-diphenyl tetrazolium (MTT) bromide and Dulbecco's modified Eagle's medium (DMEM) with 4.5 g/L glucose were obtained from Sigma-Aldrich Química S. A. Reagents used in cell culture, including nonessential amino acids (NEAA), heat inactivated fetal bovine serum (FBS), 0.05% trypsin/1 mM EDTA, antibiotic (10000 U/mL penicillin, 10,000 μg/mL streptomycin) and Hank's balanced salt solution (HBSS) were purchased from Gibco Laboratories. Dimethylsulfoxide (DMSO-*d6*), absolute ethanol and acetic acid were obtained from Merck. All reagents were of analytical grade or of the highest grade available.

#### Evaluation of acetyl and butyrylcholinesterase inhibitory activity

The AChE and BChE inhibitory activities of the compounds under study were evaluated following Ellman's method (Ellman et al., 1961; Di Giovanni et al., 2008). The solutions of enzyme (AChE, 0.5 U/mL; BChE, 0.25 U/mL) and DTNB (2.14 mM) were prepared in sodium phosphate buffer. ATCI (8.04 mM) or BTCI (5.12 mM) solutions were prepared in deionised water (conductivity < 0.1 μS·cm^−1^).

Briefly, 100 μL of sodium phosphate buffer, 40 μL of DTNB, 20 μL of the test compounds/standard inhibitors and 20 μL of AChE or BChE were incubated for 5 min at 30°C in a 96-well microplate (BRANDplates, pureGradeTM, BRAND GMBH, Wertheim, Germany). After that, 20 μL of ATCI or BTCI was added, respectively. The absorbance values were registered every minute for 5 more minutes at 30°C (at 412 nm). A control using 20 μL of sodium phosphate buffer instead of compound solution was also performed. The IC_50_ values were determined in triplicate from the dose-response inhibition curves and are expressed as mean ± standard deviation (SD).

##### Evaluation of enzyme (AChE and BChE) kinetics.

To determine the steady-state kinetic parameters (*K*_*m*_, Michaelis constant and *V*_*max*_, maximum reaction rate) of AChE and BChE, their enzymatic activities were evaluated (under the experimental conditions described above) in the presence of different ATCI or BTCI concentrations. Under our experimental conditions, AChE displayed a *K*_*m*_ of 117.3 ± 29.7 μM and a *V*_*max*_ of 0.27 ± 0.03 ΔA/min whereas BChE showed a *K*_*m*_ of 244.4 ± 15.0 μM and a *V*_*max*_ of 0.2772 ± 0.0007 ΔA/min (determined in triplicate).

##### Evaluation of AChE- and BChE-inhibitor kinetics

To evaluate the mechanism of AChE and BChE inhibition of the most promising compounds substrate-dependent kinetic experiments were performed. The catalytic rates of AChE were measured at five different concentrations of ATCI substrate (50–800 μM) and BChE at four different concentrations of BTCI substrate (62.5–500 μM) in the absence or presence of the selected inhibitors (compounds **18** and **AntiOxBEN**_**1**_) and standard inhibitor (donepezil, at concentrations between 12.5 nM and 40.0 μM). The results are presented as double reciprocal Lineweaver-Burk plots (1/V vs. 1/[S]). The kinetic data, namely *K*_*m*_ and *V*_*max*_, were acquired employing Michaelis-Menten equation. The *Ki* values were estimated using Dixon plots, by replotting the slope of each Lineweaver-Burk plot vs. the inhibitor concentration. In the Dixon plots, the *Ki* value was obtained from the x-axis intercept (–*K*_*i*_). The enzymatic reactions and measurements were performed using the same AChE and BChE assay conditions as described above (determined in triplicate). Linear regression analysis was performed using GraphPad Prism 5.0 (GraphPad Software, Inc.).

#### Evaluation of cytotoxicity/antioxidant outline in cell-based assays

##### Cell culture

SH-SY5Y (ATCC, Manassas, VA, USA), a neuroblastoma cell line, was routinely cultured in 75-cm^2^ flasks (Corning Costar, Corning, NY, USA) using DMEM with 4.5 g/L glucose, supplemented with 10% heat-inactivated FBS (v/v), 1% NEAA (v/v), 100 U/mL penicillin, and 100 μg/mL streptomycin. The cells used for all the experiments were taken between the 37th and 45th passages, to avoid phenotypic changes. For all experiments, undifferentiated SH-SY5Y cells were seeded onto 96-well plates (8,000 cells/well) in cell culture medium supplemented with 0.1% of retinoic acid for 3 days at 37°C for differentiated cells. Three days after seeding, the medium of cells was changed, adding cell culture medium with 0.1% of TPA for more 3 days before treatment.

Human hepatocellular carcinoma cells (HepG2, ECACC, UK) were also used in this study. Cells were cultured in low-glucose medium (5 mM) composed by Dulbecco's modified Eagle's medium (DMEM, D5030) supplemented with sodium pyruvate (0.11 g/L), sodium bicarbonate (3.7 g/L), HEPES (1.19 g/L), 6 mM glutamine and 10% fetal bovine serum (FBS) and 1% of antibiotic penicillin-streptomycin 100 × solution. HepG2 (2.5 × 10^4^ cells/well) cells were seeded in a 96-well plate and proliferate for 24 h before treatment.

All cells were maintained and cultured at 37°C in a humidified atmosphere of 95% air/5% CO_2_ and passaged weekly by trypsinization (0.05% trypsin/1 mM EDTA) when reaching 70–80% confluence.

##### Evaluation of cytotoxicity

The cytotoxicity profile of the compounds under study was evaluated in differentiated SH-SY5Y cells (8,000 cells/well), according the differentiation protocol previously described, and in HepG2 cells (2.5 × 10^4^ cells/well), seeded into 96-well plates. Then, the cells were exposed to increased concentrations of the test compounds (1, 10 and 50 μM) in cell culture medium for 24 h in SH-SY5Y cells or 48 h in HepG2 cells and the cytotoxicity was evaluated through measuring changes in cellular metabolic activity using the MTT or resazurin reduction assays, respectively (Silva et al., 2016). In both assays, the reduction of MTT tetrazolium salt or resazurin to MTT formazan or resorufin, respectively, by cellular dehydrogenases present in viable cells, gives insights on cell metabolic activity.

For the MTT assay, the cell culture medium was removed after the incubation time, followed by the addition of fresh cell culture medium containing 0.5 mg/mL MTT and incubation for 30 min. After this incubation period, the cell culture medium was removed, and the formed formazan crystals dissolved in DMSO. The absorbance was measured at 550 nm in a multi-well plate reader (BioTek Instruments, Vermont, USA). The percentage of MTT reduction relative to that of the control cells was used as the cytotoxicity measure [MTT reduction (% of control)] as means ± SEM of four independent experiments.

Regarding the resazurin reduction assay, after the incubation time, the medium was replaced by fresh medium containing resazurin (10 μg/mL) prepared in sterile PBS (1X) and left to react for 1 h. The fluorescent signal was monitored using a 540 nm excitation wavelength and 590 nm emission wavelength in a Cytation 3 reader (BioTek Instruments Inc., USA). The percentage of resazurin reduction relative to that of the control cells was used as the cytotoxicity measure [resazurin reduction (% of control)] as means ± SEM of four independent experiments.

Control experiments were performed for both viability endpoints by adding vehicle (medium with 0.1% DMSO) instead of the compound solution.

##### Evaluation of neuroprotective activity

The antioxidant efficiency of the new HBAc derivatives was evaluated in the presence of Aβ1–42 peptide. The synthetic peptide Aβ1–42, corresponding to neurotoxic amino acid residues of the human amyloid-beta protein (Aβ), was dissolved in sterile water in order to facilitate peptide solubilization at a concentration of 1 g/L (221.5 μM). Aβ1–42 aliquots were then stored at −20°C until use (enriched oligomeric Aβ1–42 preparation).

Undifferentiated SH-SY5Y cells were seeded onto 96-well plates (8000 cells/well) and differentiated as previously described. The cells were incubated with **AntiOxBEN**_**1**_, **AntiOxBEN**_**2**_ and **15–18** (10 μM) for 24 h. Then, oligomeric Aβ1–42 (25 μmol/L) was added to the culture medium of the SH-SY5Y cells for more 48 h. After incubation time, cellular metabolic activity was determined using the resazurin reduction assay as previously described.

### Data analysis

For the radical scavenging and enzymatic inhibition studies, the compounds were initially screened at 50 μM. For the most potent compounds, dose-response curves were plotted and IC_50_ values were estimated by non-linear analysis. For the cytotoxicity assays, MTT and resazurin reduction were calculated for each treatment as the % of control untreated cells and plotted in column graphs.

Data were analyzed in GraphPad Prism 5.0 software (GraphPad Software, Inc.), with all results being expressed as means ± SEM for the number of experiments indicated. In data analysis, student's *t*-test was used for comparison of two means, and one-way ANOVA with Dunnet multiple comparison post-test was used to compare more than two groups with one independent variable. Significance was accepted with ^*^P < 0.05, ^**^P < 0.01, ^***^P < 0.0005, ^****^P < 0.0001.

### Molecular docking studies

Docking simulations were performed with the Schrödinger 2017 package (Schrödinger suite 2017-2, 2017). The crystal structure of the human BChE was downloaded from the PDB (code: 4B0O) (Wandhammer et al., 2013) and pre-processed with the Protein Preparation Workflow (Schrödinger suite 2017-2, 2017). This procedure included steps such as addition of cap termini, optimization of hydrogens, protonation states of residues and H-bond network optimization. Only one water molecule stabilized through two hydrogen bonds with the protein (residues Asp70 and Ser79) was retained in the pocket for the simulations. Before docking, a receptor grid was generated using the co-crystallized ligand as a center (box length = 20 Å, *van der Waals* scaling factor = 1.0, partial charge cut-off = 0.25). Ligands were docked to the protein with Glide SP (Standard Precision) (Schrödinger suite 2017-2, 2017). The best pose according to the parameter “E_model_ energy” was retained and considered representative of the calculation. The docking protocol was validated measuring the RMSD (root mean square deviation) between 5 co-crystallized ligands downloaded from the PDB and their calculated poses extracted from docking (RMSD values: 4B0O = 1.65, 4AXB = 4.26, 4BDS = 0.37, 1P0M = 3.37, 1P0P = 2.25).

## Results and discussion

### Chemistry

**Scheme 1:**
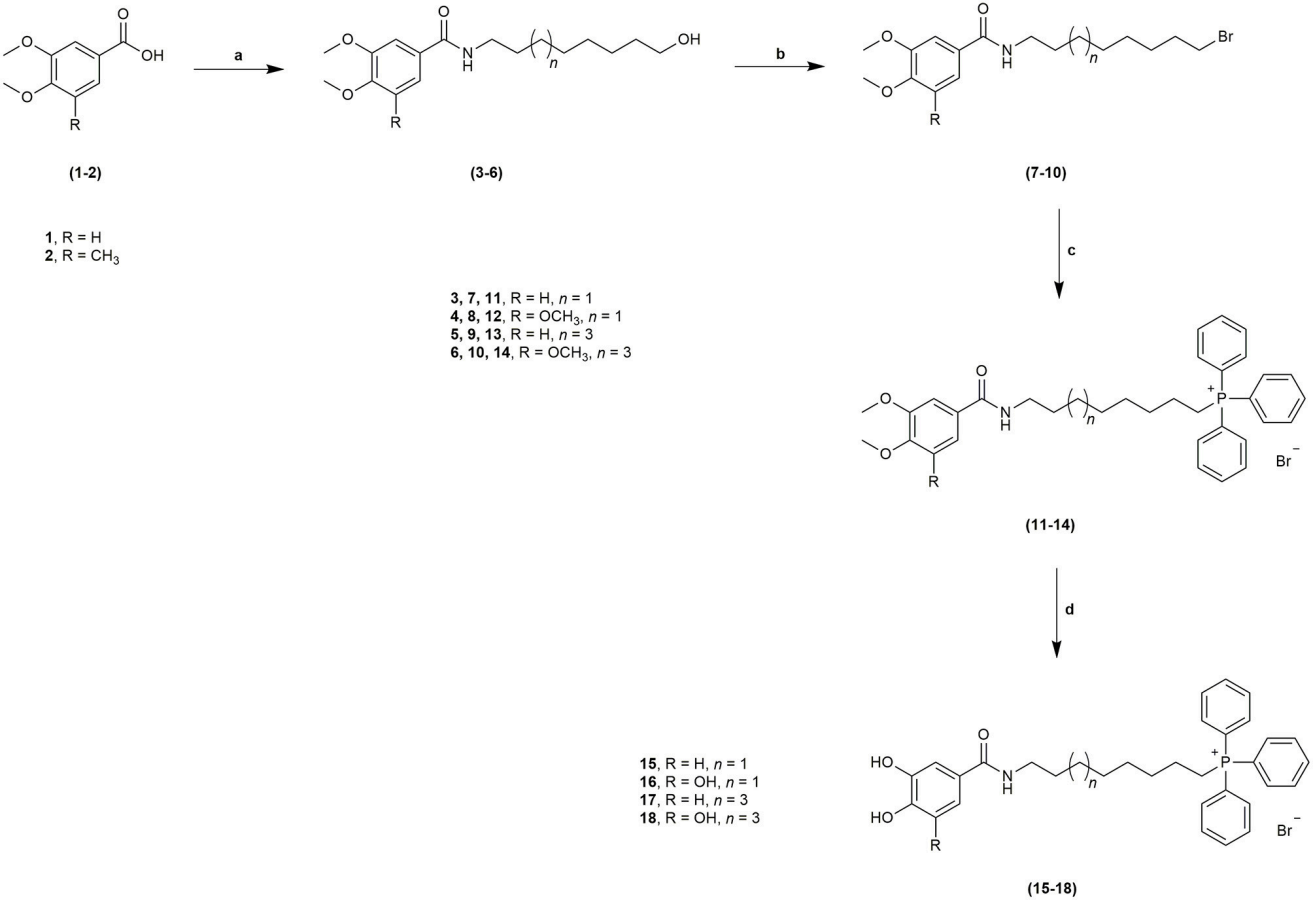
Synthetic strategy followed to obtain derivatives **15**–**18** from benzoic acids **1**–**2**. Reagents and conditions: **a**. POCl_3_, NH_2_(CH_2_)_7_CH_2_OH or NH_2_(CH_2_)_9_CH_2_OH, DIPEA, DCM, r.t., 1–2 h; **b**. C_2_Br_2_Cl_4_, diphos, THF, r.t., 20 h; **c**. PPh_3_, 100°C, 48 h; **d**. BBr_3_, anhydrous DCM, −70°C to r.t., 12 h.

The novel HBAc derivatives were obtained following a four-step synthetic strategy depicted in Scheme 1, using 3,4-dimethoxybenzoic acid (**1**) and 3,4,5-trimethoxybenzoic acid (**2**) as starting compounds. The first synthetic step (**a**) was an amidation reaction of the acids **1** and **2**: phosphorus oxychloride was used as coupling agent for the introduction of the 8-aminooctan-1-ol and 10-aminodecan-1-ol spacers leading to derivatives **3–6**. Subsequently, these derivatives were treated with 1,2-dibromotetrachloroethane and 1,2-bis(diphenylphosphine)ethane (diphos) (step **b**), leading to halogenated derivatives **7–10**. The triphenylphosphonium salts **11–14** were then obtained by a reaction with triphenylphosphine in step **c**, followed by a final *O*-demethylation process (step **d**) to afford derivatives **15**–**18**. Using this synthetic strategy, four derivatives were obtained, bearing different spacer lengths and number of phenolic functions at the aromatic ring.

The structural identity was confirmed by nuclear magnetic resonance (NMR) spectroscopy (^1^H NMR, ^13^C NMR and DEPT135) and electron impact (EI/MS) or electrospray ionization mass spectra (ESI/MS).

### Evaluation of radical scavenging activity

Total antioxidant capacity assays (DPPH^•^ and ABTS^•+^) were used to evaluate the antioxidant capacity of compounds **15**–**18**. In these assays, the ability of an antioxidant to transfer a hydrogen atom or an electron to a stable free radical is related with a radical absorbance decrease and, consequently, compounds with a higher antioxidant activity display a lower IC_50_ value. The data related with **AntiOxBEN**_**1**_ and **AntiOxBEN**_**2**_ (Figure 1) was previously published, and included for comparative analysis (Teixeira et al., 2017b).

Results showed that pyrogallol derivatives **AntiOxBEN**_**2**_, **16**, **18** displayed a superior antioxidant activity over the corresponding catechols **AntiOxBEN**_**1**_, **15**, **17** (Table 1). Although the length of the carbon alkyl chain did not alter the antioxidant capacity of the synthesized HBAc derivatives, the presence of the TPP^+^ moiety decreased their antioxidant capacity, a fact that may be related with stereochemical hindrances.

**Table 1:** Total antioxidant capacity and ChEs inhibitory activity (IC_50_) data of compounds **AntiOxBEN**_**1**_, **AntiOxBEN**_**2**_, **15–18** and donepezil.

**Compound**	**Structure**	**IC**_**50**_ **(**μ**M** ± ***SD*****)**			**IC_50_ (nM ± SD)**	**SI**	***E*_p_ (mV)**
		**DPPH**^•^	**ABTS**^•+^	**AChE**	**BChE**		
**AntiOxBEN**_**1**_	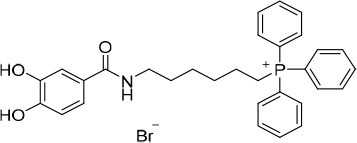	31.0 ± 0.8^*^	20.7 ± 0.8^*^	>50	85 ± 5	> 588	219^*^
**AntiOxBEN**_**2**_	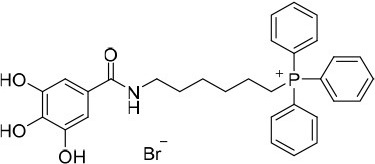	14.0 ± 0.3^*^	6.5 ± 0.5^*^	40.5 ± 7.0	474 ± 17	86	115^*^
**15**	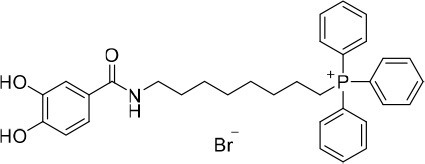	31.2 ± 0.9	23.5 ± 1.3	11.2 ± 0.8	106 ± 5	106	216
**16**	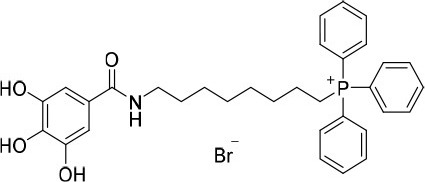	16.8 ± 1.6	6.8 ± 0.6	15.7 ± 0.9	255 ± 9	62	121
**17**	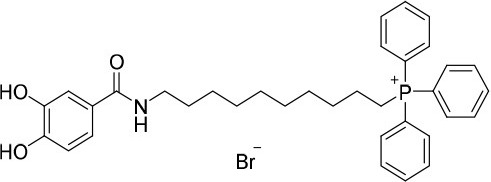	31.0 ± 0.6	19.4 ± 1.0	7.7 ± 0.4	195 ± 20	41	210
**18**	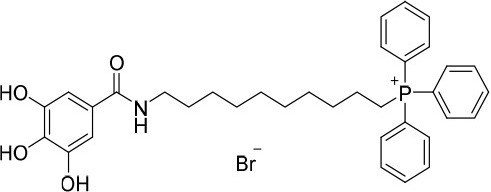	10.2 ± 1.1	7.4 ± 0.5	7.2 ± 0.5	553 ± 22	13	114
**Donepezil**	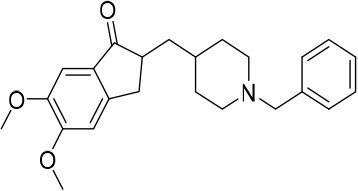	n.d.	n.d.	0.025 ± 0.001	2200 ± 200	0.011	n.d.

*n.d.: not determined*.

* *Data from Teixeira et al. (2017a)*.

SI: BChE selectivity index = IC_50(AChE)_/IC_50(BChE)._

### Evaluation of redox properties

During the last decade, electrochemical methods have attracted a great deal of attention given their enormous potential for the assessment of antioxidant capacity. Since antioxidants can act as reducing agents, they are electrochemically active and can be measured using electrochemical techniques (Teixeira et al., 2017a,b; Apak et al., 2018). Moreover, these methods provide a fast, simple and a low-cost alternative to measure antioxidant compounds and monitor their antioxidant capacity in biological and food samples (Teixeira et al., 2017a,b; Apak et al., 2018).

The oxidative profile of compounds **15**–**18** was evaluated at physiological pH 7.4, by differential pulse and cyclic voltammetry, using a glassy carbon working electrode. The DPV voltammograms obtained for catechol derivatives (**15** and **17**) showed the occurrence of only one anodic peak at an oxidation potential (*E*_p_) between + 210 and + 216 mV (Table 1) at physiological pH. Cyclic voltammograms were also recorded for these derivatives and the data obtained was characteristic of an electrochemical reversible reaction showing only one anodic peak and one cathodic peak on the reverse scan. DPV of the compounds containing a pyrogallol moiety (**16** and **18**) were also obtained and showed the presence of two overlapped anodic waves occurring at an *E*_p_ ranging from + 114 to + 121 mV, at physiological pH (Table 1). The cyclic voltammograms also showed the presence of two overlapped peaks with no reduction peak on the cathodic sweep, indicating that the oxidative process may correspond to an irreversible reaction.

The results were in accordance with the data previously obtained for **AntiOxBEN**_**1**_, **AntiOxBEN**_**2**_ and their parent HBAc (PA and GA) (Teixeira et al., 2017b) reinforcing our believe that the redox potential of these mitochondriotropic antioxidants is mainly associated with the number of hydroxyl substituents on the aromatic pattern. Furthermore, the data showed that the oxidation potentials observed are closely related to the antioxidant activity evaluated by total antioxidant assays. In fact, pyrogallol derivatives that presented lower oxidation potentials, exhibited higher antioxidant activities when compared with compounds bearing a catechol group (Table 1). On the other hand, the results showed that the increase of the length of alkyl spacer does not significantly influence the redox properties of the compounds, under the experimental conditions used.

### Evaluation of AChE/BChE inhibitory activities

The inhibitory activity of the compounds **AntiOxBEN**_**1**_, **AntiOxBEN**_**2**_, and **15**–**18** on AChE and BChE was evaluated by the Ellman's method (Ellman et al., 1961; Di Giovanni et al., 2008). ATCI and BTCI were used as substrates for AChE or BChE, respectively, generating thiocholine and acetate or butyrate. In this assay, the released thiocholine reacts with the ion DTNB to produce the anion 5-thio-2-nitrobenzoate (TNB^2−^), which was determined by UV/Vis spectroscopy (Ellman et al., 1961; Di Giovanni et al., 2008), enabling the determination of the inhibitory potency (IC_50_ values) after incubation with the test compounds (Table 1). Donepezil (Table 1) a selective and reversible AChE inhibitor drug approved by FDA for AD treatment (Colović et al., 2013) was used as standard.

With the exception of **AntiOxBEN**_**1**_, which did not have any effect on AChE at 50 μM, all derivatives effectively inhibited both AChE and BChE under the experimental conditions. Concerning AChE, derivatives presenting the longer spacers showed improved binding to the enzyme's active site. In fact, compounds **17** and **18**, with a ten-carbon chain, showed greater potency (IC_50_ = 7.7 and 7.2 μM, respectively) than **AntiOxBEN**_**1**_, **AntiOxBEN**_**2**_ (IC_50_ = > 50 and 40.5 μM, respectively) and derivatives **15–16** (IC_50_ = 11.2 and 15.7 μM, respectively) (Table 1). The compounds with shorter alkyl spacers (**AntiOxBEN**_**1**_, **15** and **16**) showed higher inhibitory potency toward BChE (IC_50_ = 85, 106 and 255 nM, respectively).

Catechol derivatives **AntiOxBEN**_**1**_, **15** and **17** (IC_50_ = 85–190 nM) showed better performance than pyrogallols **AntiOxBEN**_**2**_, **16** and **18** (IC_50_ = 255–550 nM).

As expected donepezil showed a higher potency for AChE than BChE (IC_50_ = 25 ± 1 nM and 2.2 ± 0.2 μM, respectively).

The BChE selectivity index (SI) calculated from the ratio of the IC_50_ values of AChE and BChE showed that the most selective compounds were **AntiOxBEN**_**1**_ and **15** (over 588- and 106-fold more active toward BChE than AChE, respectively). Thus, the modifications performed in PA significantly increased their inhibitory effect in BChE.

### Evaluation of drug-like properties

The drug-like properties were determined for compounds **AntiOxBEN**_**1**_, **AntiOxBEN**_**2**_, **15**–**18**, donepezil and precursors PA and GA. The calculated physicochemical parameters included: molecular weight (MW), partition coefficient (clog P), topological polar surface area (tPSA in Å^2^), number of hydrogen acceptors (HBA), number of hydrogen donors (HBD), number of rotatable bonds (*n*rotb) and blood (plasma)-brain partitioning (logBB) (Table 2). Along theoretical calculations of clogP, it was observed that the programs used do not include ionic compounds (as mitochondriotropic salts). Therefore, all the calculations were done with the neutral molecule, resulting in higher partition coefficients than expected. Due to the high volume of the aliphatic TPP^+^ moiety, the MW and clogP values overcome the upper limit established by the “Rule of 5” (MW ≤ 500 g/mol and clogP ≤ 5) (Lipinski et al., 2001). However, this problem can be counterbalanced by its therapeutic profile, i.e., the ability of TPP^+^ moiety to rapidly cross phospholipid bilayers and, consequently, accumulate in mitochondria (Murphy and Smith, 2007).

**Table 2:** Drug-like properties of compounds AntiOxBEN_1_, AntiOxBEN_2_, 15-18 and donepezil.

**Compound**	**MW^a^**	**clog P^a^**	**tPSA (Å^2^)^a^**	**HBA^a^**	**HBD^a^**	***n*rotb^a^**	**log BB^a^**
**AntiOxBEN**_**1**_	498.6	6.49	69.56	4	3	12	−0.588
**AntiOxBEN**_**2**_	514.6	5.81	89.79	5	4	12	−0.665
**15**	526.6	6.93	69.56	4	3	14	−0.594
**16**	542.6	6.31	89.79	5	4	14	−0.675
**17**	554.7	7.32	69.56	4	3	16	−0.600
**18**	570.7	6.74	89.79	5	4	16	−0.685
**Donepezil**	379.5	3.54	38.77	4	0	6	0.790
**CNS**^+^ **drugs** (Pajouhesh and Lenz, 2005; Hitchcock and Pennington, 2006; Fong, 2015; Rankovic, 2015; Nikolic et al., 2016)	< 450	< 5	< 60–70	< 7	< 3	< 8	≥−1

a *Properties calculated using StarDrop software. MW, molecular weight; clogP: logarithm of the octanol-water partition coefficient; tPSA, topological polar surface area; HBA, number of hydrogen acceptors; HBD, number of hydrogen donors; nrotb, number of rotatable bonds; log BB, logarithm of the ratio of the concentration of a drug in the brain and in the blood*.

The values of HBD and HBA obtained were in accordance with the general drug-likeness requirements of the Linpinski's “Rule of 5” (with HBD < 5 and HBA < 10) (Lipinski et al., 2001).

Another key measurement is the prediction of blood-brain barrier (BBB) permeability, determined by the logBB, which is the ratio of the steady-state concentrations of the drug in the brain and in the blood. Compounds with logBB < −1 are poorly distributed to the brain and are unlikely to function as effective central nervous system (CNS) drugs (Clark, 1999). Compounds **AntiOxBEN**_**1**_, **AntiOxBEN**_**2**_, and **15**–**18** displayed logBB ≥ −1, pointing toward potential BBB permeability.

As expected, the values obtained for donepezil were all in accordance with the general drug-likeness requirements of the Linpinski's “Rule of 5.”

### Evaluation of cytotoxicity profile

**Figure 2:**
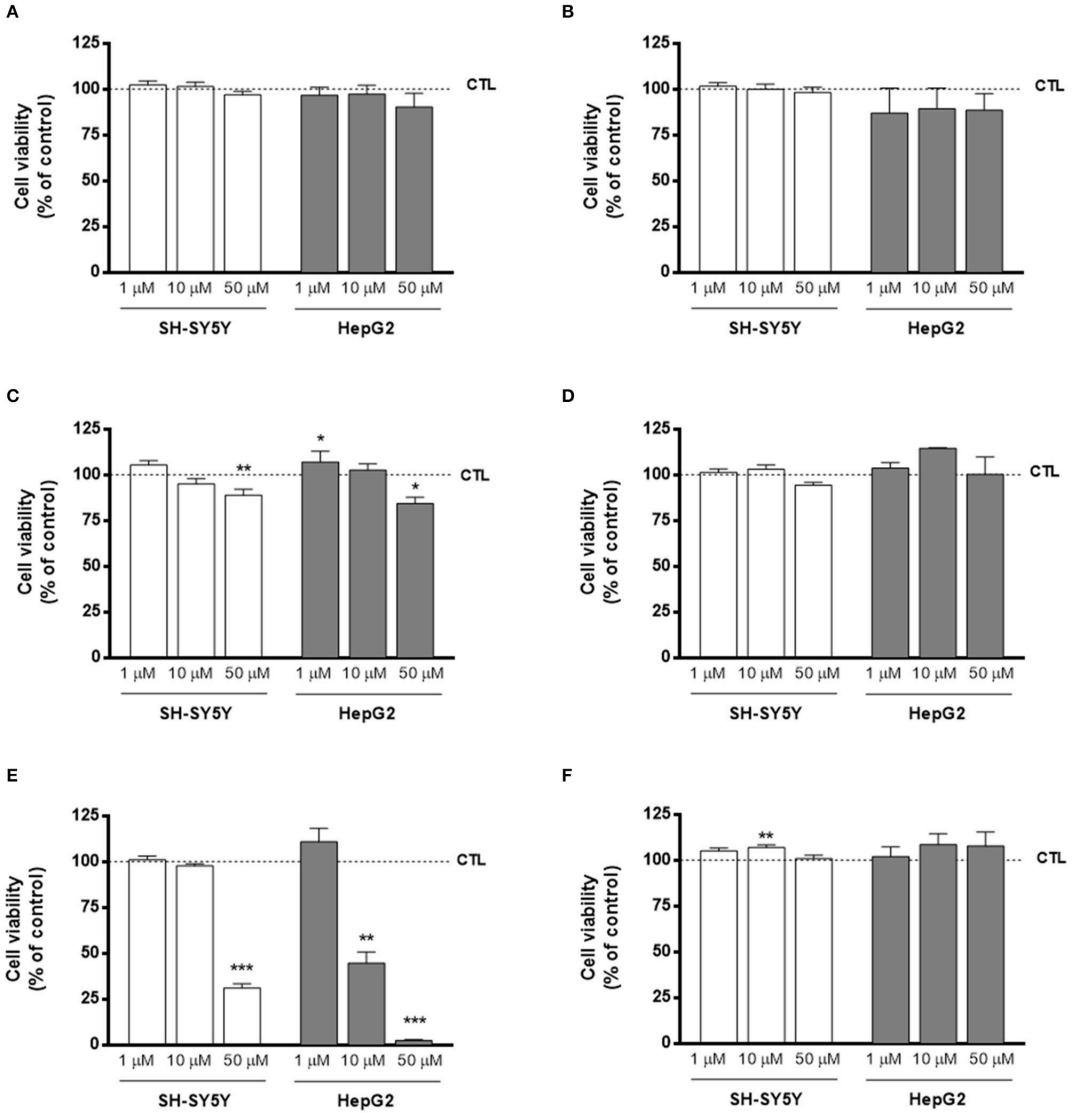
Cytotoxicity profile of compounds **(A) AntiOxBEN**_**1**_, **(B) AntiOxBEN**_**2**_, **(C) 15**, **(D) 16**, **(E) 17**, and **(F) 18** measured by changes in cellular metabolic activity on human neurablastoma SH-SY5Y and human hepatocarcinoma HepG2 cells after 24 or 48 h treatment time, respectively, at three different concentrations (1, 10, and 50 μM). Cellular viability was evaluated using two methods: MTT in differentiated SH-SY5Y and resazurin reduction assays in HepG2 cells. Untreated cells were used as control. Data are means ± SEM of three independent experiments and the results are expressed as percentage of control (control = 100%), which represents the cell density without any treatment in the respective time point. Significance was accepted with ^*^P < 0.05, ^**^P < 0.01, ^***^P < 0.0005 vs. control.

The cytotoxicity profile of compounds **AntiOxBEN**_**1**_, **AntiOxBEN**_**2**_, and **15**–**18** was evaluated by the determination of the cellular metabolic activity in differentiated human neuroblastoma cells (SH-SY5Y) and human hepatocarcinoma cells (HepG2), after a 24 or 48 h incubation period, respectively, at three different concentrations (1, 10, and 50 μM) (Figure 2). Both cell lines are often used in the preclinical safety assessment of drug candidates, being the SH-SY5Y cells of particular interest concerning the drug candidates for CNS (Lin and Will, 2012). Metabolically active cells reduce MTT and resazurin to formazan and resorufin, respectively, which can be spectrophotometrically quantified, providing an indirect measure of cell viability (Lobner, 2000). Although the **AntiOxBEN**_**1**_, **AntiOxBEN**_**2**_ cytotoxicity on HepG2 has been previously described (Teixeira et al., 2017b), they were re-evaluated for comparative analysis.

In general, the cytotoxicity profile of all compounds followed the same trend independently of the cell line used for safety assessment. Moreover, the results clearly showed that compounds **AntiOxBEN**_**2**_, **16** and **18**, containing the pyrogallol moiety, did not alter cellular metabolic activity for all tested concentrations, presenting a larger safety margin toward SH-SY5Y and HepG2 cells. Interestingly, compound **18** slightly increased SH-SY5Y (1 and 10 μM) and HepG2 (10 and 50 μM) metabolic activity.

On the other hand, compounds with the catechol moiety (**AntiOxBEN**_**1**_, **15** and **17**) presented dose-dependent toxicity in both SH-SY5Y and HepG2 cells. Compounds **15** and **17** were the most cytotoxic compounds, showing that longer spacers increased the toxicity effects of catechol-derived compounds.

From the cytotoxic profile on both SH-SY5Y and HepG2 cells, it can be concluded that compounds containing the catechol moiety, with exception of **AntiOxBEN**_**1**_, exhibited a higher toxicity than compounds with the pyrogallol moiety.

### Evaluation of neuroprotective properties

Aβ aggregation, the pathological hallmark of AD, is the most targeted biomarker in drug discovery and development for AD. As ChEs colocalize with the amyloid and may contribute to the generation of amyloid proteins, it was found relevant to check the neuroprotective properties of compounds under study. The experiments were performed in SH-SY5Y cells using Aβ1–42, a synthetic toxic fragment of the amyloid protein (Resende et al., 2008), as oxidative insult. Consequently, SH-SY5Y cells were treated with Aβ1–42 peptide (25 μM) for 48 h and then the cell metabolic activity was measured by colorimetric resazurin assay, which significantly decrease the cell viability of about 28% when compared to control.

**Figure 3:**
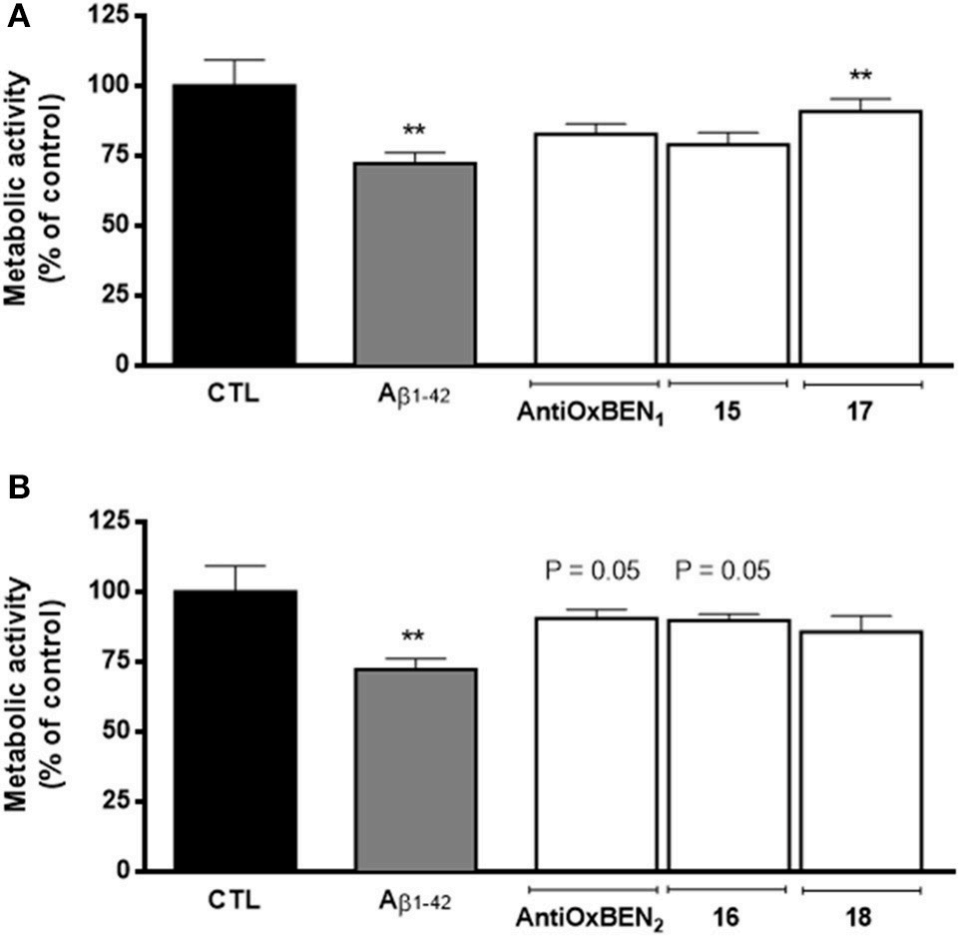
Neuroprotective effects of compounds **(A) AntiOxBEN**_**1**_, **15** and **17** and **(B) AntiOxBEN**_**2**_, **16** and **18** in human neuroblastoma SH-SY5Y cells against Aβ1–42 peptide-induced cytotoxicity measured by changes in cellular metabolic activity. Cells were pre-treated with mitochondriotropic antioxidants (10 μM) for 24 h before treatment with Aβ1–42 peptide (25 μM) for 48 h more. The comparisons were performed by using one-way ANOVA between the control (Aβ1–42 peptide) vs. preparation where antioxidants **AntiOxBEN**_**1**_, **AntiOxBEN**_**2**_ and **15**–**18** were pre-incubated. Data are means ± SEM of three independent experiments and the results are expressed as percentage of control (control = 100 %), which represents the cell density without any treatment in the respective time point. Significance was accepted with ^**^P < 0.01 vs. control.

In general, pre-treatment of SH-SY5Y cells with compounds **AntiOxBEN**_**1**_, **AntiOxBEN**_**2**_ and **15**–**18** (10 μM, Figure 3) for 24 h reduced Aβ1–42 peptide-induced cytotoxicity, being those effects more evident for compounds **17** (presenting a catechol moiety and a longer spacer) and **AntiOxBEN**_**2**_ and **16** (with a pyrogallol moiety and shorter alkyl spacers) (Figure 3). Several mechanisms, such as inhibition of mitochondrial Ca^2+^ overload and of caspase cascade, may underlie HBAc derivatives-induced neuroprotection against Aβ neurotoxicity, which will be the subject of a follow-up study.

### Evaluation of enzyme-inhibition mechanism of AntiOxBEN_1_ and 18

**Figure 4:**
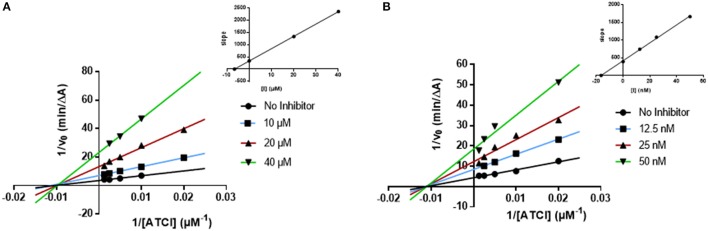
Kinetic studies on the mechanism of AChE inhibition by compounds and **(A)** 18, and **(B)** donepezil. The effect of the inhibitors on the enzyme was determined from the double reciprocal plot of 1/rate (1/V) vs. 1/substrate concentration in presence of varying concentrations of the inhibitors. The *Ki* values were calculated by the intersection of the curves obtained by plotting 1/V vs. the inhibitor concentration for each substrate concentration (Dixon plots insets on the top right).

**Figure 5:**
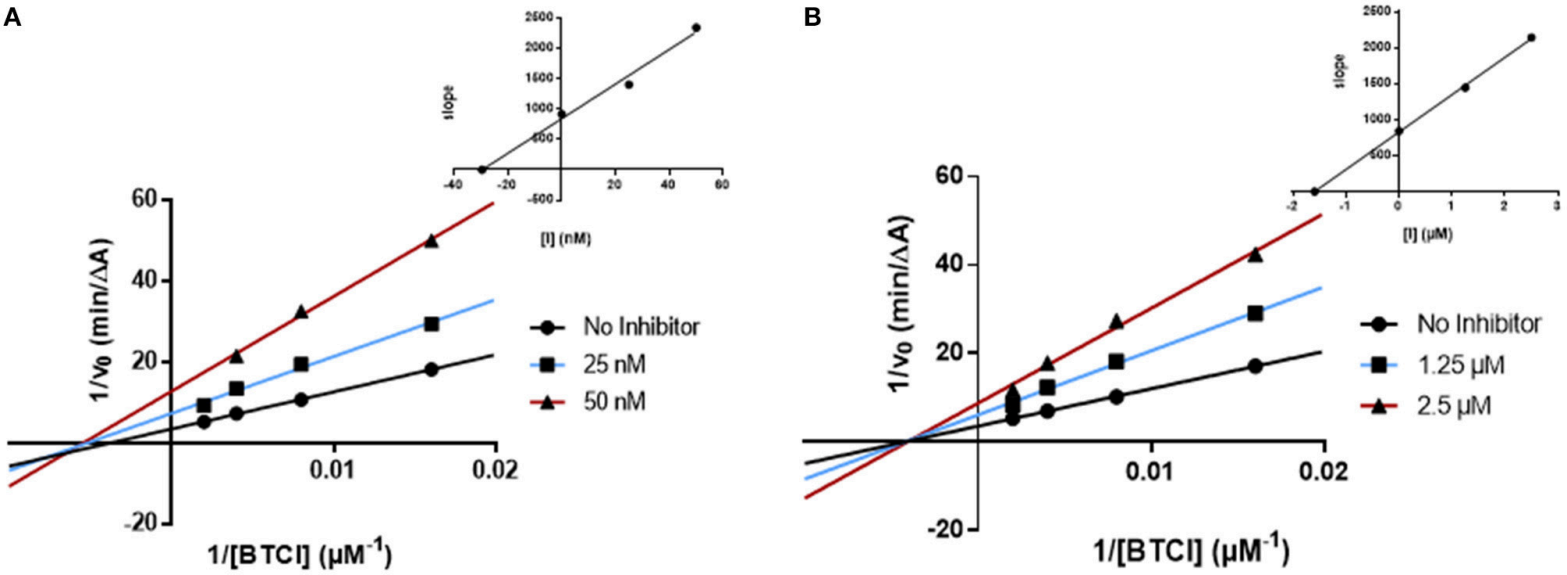
Kinetic studies on the mechanism of BChE inhibition by **(A)** AntiOxBEN_1_ and **(B)** donepezil. The effect of the inhibitors on the enzyme was determined from the double reciprocal plot of 1/rate (1/V) vs. 1/substrate concentration in presence of varying concentrations of the inhibitors. The *Ki* values were calculated by the intersection of the curves obtained by plotting 1/V vs. the inhibitor concentration for each substrate concentration (Dixon plots insets on the top right).

In order to understand the AChE and BChE inhibitory mechanism of the most promising ChE inhibitors, kinetic experiments were performed. For this purpose, the enzymatic inhibition kinetics was measured, using different substrate concentrations in absence or presence of the selected compounds at different concentrations, including the standard inhibitor donepezil (Figures 4, 5). Graphical analysis of the reciprocal Lineweaver–Burk plots was used to determine Michaelis-Menten reaction kinetic parameters (*K*_*m*_ and *V*_*max*_).

#### Ache kinetics

Concerning compound **18** (Figure 4A), results showed that *V*_*max*_ decreased while *K*_*m*_ remained unchanged. The same kinetic behavior was observed for donepezil (Figure 4B) (Sugimoto et al., 2002; Pohanka, 2014). The Lineweaver-Burk plots obtained for compound **18** and donepezil displayed a series of converging lines on the same point of the x-axis (1/[S]), profiling a non-competitive inhibition mechanism (Figure 4).

From the Dixon plots, obtained from the replots of the slopes of the Lineweaver–Burk plots vs. inhibitor concentrations (Figure 4, upper right corners), the AChE inhibition binding affinities, determined as inhibition constants (*Ki*), were calculated. Compound **18** (Figure 4A) displayed a *Ki* value of 6.6 μM, which correlated well with its experimental IC_50_ (7.2 μM). The similar IC_50_ and *Ki* values confirmed its non-competitive inhibition profile. The kinetic inhibition profile is similar to donepezil (*Ki* = 16.4 nM and IC_50_ = 24.6 nM, Figure 4B).

#### BChE kinetics

The kinetics determined for **AntiOxBEN**_**1**_ (the most selective compound) showed that the *V*_*max*_ decreased while *K*_*m*_ remained unchanged (Figure 5A). The same profile was obtained for donepezil (Figure 5B). The Lineweaver-Burk plots obtained for **AntiOxBEN**_**1**_ and donepezil (Figures 5A,B) displayed a series of converging lines on the same point of the x-axis (1/[S]), profiling a non-competitive inhibition mechanism.

From the Dixon plots, obtained from the replots of the slopes of the Lineweaver–Burk plots vs. inhibitor concentrations (upper right corner), the BChE inhibition binding affinities, determined as inhibition constants (*Ki*), were calculated. **AntiOxBEN**_**1**_ (Figure 5A) displayed *Ki* value of 29.5 nM and IC_50_ = 85 nM. This compound displayed IC_50_ and *Ki* values different but in the same range, with an acceptable correlation between its respective values. Donepezil showed a similar kinetic behavior (*Ki* = 1.6 μM and IC_50_ = 2.2 μM, Figure 5B).

### Molecular docking studies

Molecular docking simulations of the best BChE inhibitors of the series were performed to study the main ligand-protein interactions. In the modeling, the crystallized human protein structure 4B0O (PDB code) was used. (Wandhammer et al., 2013). The ligands were docked with Glide SP mode (Schrödinger suite 2017-2, 2017) and the best pose according to the energetic parameter E_model_ was retained for graphical purposes (see Table 3 with SP and E_model_ values). The geometrical quality of the docking protocol was evaluated measuring the RMSD between co-crystallized ligands and theoretical poses extracted from docking (see Methods).

**Table 3:** E_model_ and SP scoring values for co-crystallized ligands and our set of derivatives.

**Compound**	**E_model_**	**SP score**
4B0O^*^	−64.70	−7.81
4AXB^*^	−36.09	−6.97
4BDS^*^	−62.52	−8.18
1P0M^*^	−34.03	−5.07
1P0P^*^	−44.67	−5.01
**AntiOxBEN**_**1**_	−104.97	−8.58
**AntiOxBEN**_**2**_	−120.22	−9.85
**15**	−109.56	−9.58
**16**	−114.26	−9.57
**17**	−111.74	−9.46
**18**	−106.26	−8.51

* *Co-crystallized ligands. Units, kcal/mol*.

The RMSD values were lower than 2.5 Å for 3 out of 5 co-crystallized ligands showing the ability of the docking to reproduce some co-crystallized structures. For instance, the co-crystallized ligands in 4BDS (Nachon et al., 2013) and 4B0O (Wandhammer et al., 2013) were retrieved with a RMSD of 0.37 and 1.65 Å respectively. In agreement with the crystallized structure, the binding mode retrieved by the ligand tacrine in 4BDS located the heterocyclic scaffold near the residue Trp82 and established π-π stacking interactions with the residue. In the 4B0O structure, the pose described by docking retrieved the π-π stacking interactions between the benzylpyridinium group of the ligand and residues Trp82 and Tyr332. In addition, the trichloroacetimidate is oriented toward the same area in the receptor close to residues Thr120 and Ser287.

**Figure 6:**
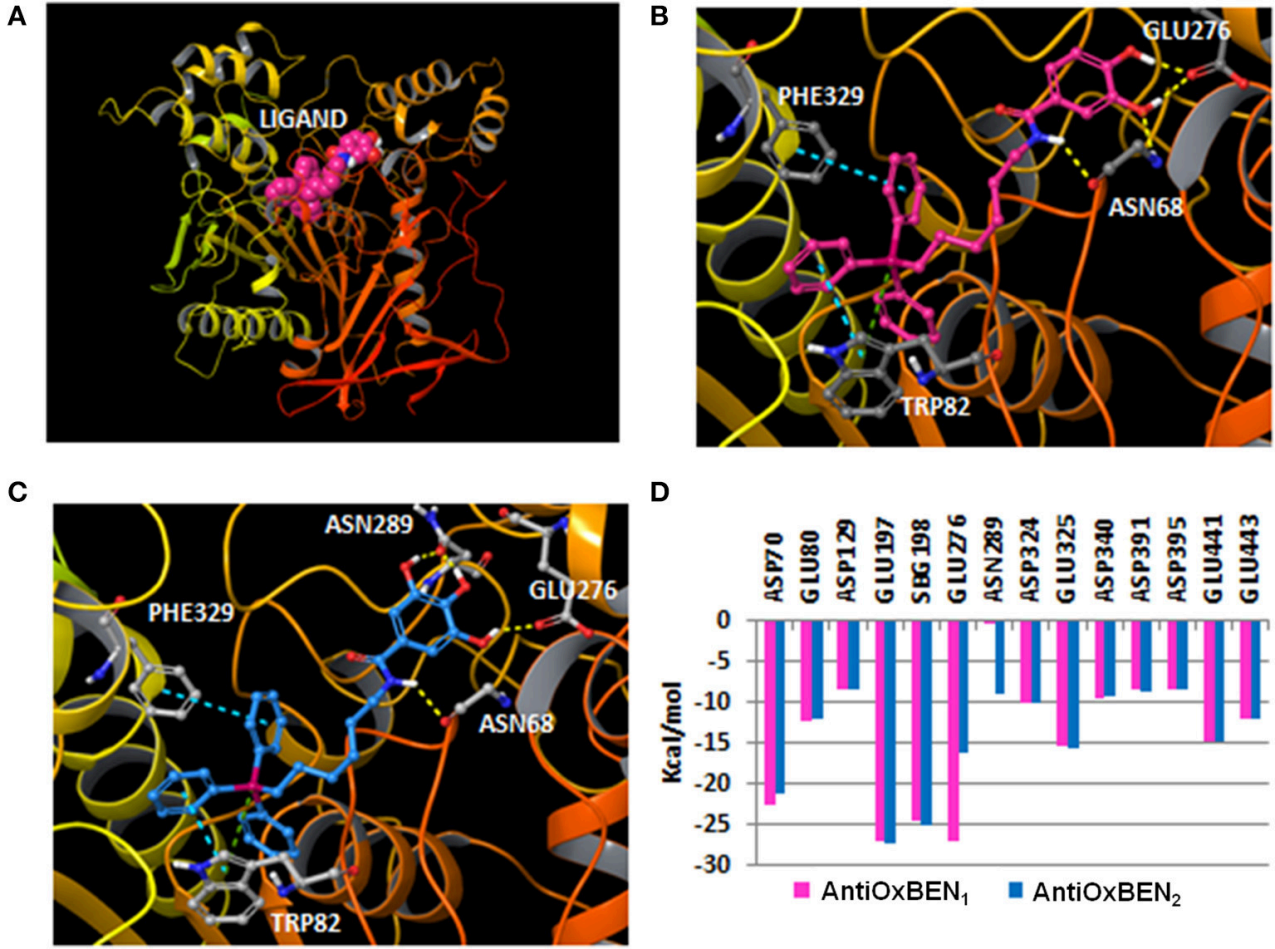
**(A)** General perspective of **AntiOxBEN**_**1**_ (CPK representation) bound to the BChE (ribbons) extracted from molecular docking; **(B)** Binding mode calculated with molecular docking for **AntiOxBEN**_**1**_ (pink carbons) in the BChE (color code: yellow dashes for hydrogen bonds, blue dashes for π-π stacking interactions and green dashes for π-cation interactions); **(C)** Pose yielded by docking for **AntiOxBEN**_**2**_ (blue carbons) in the BChE; and **(D)** Coulomb residue contributions to the binding between the BChE and **AntiOxBEN**_**1**_ and **AntiOxBEN**_**2**_.

After docking simulations, all the compounds in our series yielded a similar binding mode that directed the triphenylphosphonium group toward the bottom of the pocket, close to the catalytic triad characterized by residues Ser198, Glu325 and His438, whereas the hydroxybenzamide was positioned toward the surface of the cavity (Figure 6). The most active compound in the series (**AntiOxBEN**_**1**_) established a hydrogen bond between the nitrogen of the amide group and the residue Asn68. The binding mode also yielded additional hydrogen bonds with residues Asn68 and Glu276 by anchoring with the hydroxyl groups of the phenyl ring. Additionally, **AntiOxBEN**_**1**_ established π-π stacking and π-cation interactions between the triphenylphosphonium group and residues Phe329 and Trp82. The residue Trp82 in the anionic site has been previously described as a key residue that establishes π-cation interactions with choline scaffolds (Nicolet et al., 2003; Bacalhau et al., 2016). A similar binding mode was detected for **AntiOxBEN**_**2**_ with three hydroxyl groups in the phenyl ring. The introduction of a third hydroxyl group in the phenyl ring originated an additional hydrogen bond with the residue Asn289 (Figure 6). However, the hydroxyphenyl ring is slightly shifted toward the residue Asn289 causing the disruption of the hydrogen bond with residue Asn68. This fact could be related with the reduction of inhibitory activity shown by **AntiOxBEN**_**2**_ in comparison to **AntiOxBEN**_**1**_. Moreover, Coulomb interactions could play an important role in the ligand-protein recognition since there are different charged residues in the protein pocket and the studied compounds bear a positive charge in the phosphonium group. We calculated the Coulomb residue contributions to the binding for **AntiOxBEN**_**1**_ and **AntiOxBEN**_**2**_. The key contribution of some residues to the binding is represented in Figure 6D. Some residues showed important contributions, such as Glu276, Glu197, SBG198 (conjugated Ser in the aged enzyme), and Asp70. **AntiOxBEN**_**1**_ and **AntiOxBEN**_**2**_ presented some differences in the Coulomb energies for residues Glu276 and Asn289. While **AntiOxBEN**_**2**_ showed a noticeable reduction of Coulomb interactions with residue Glu276 compared to **AntiOxBEN**_**1**_, the interactions with the residue Asn289 were higher. The introduction of additional hydroxyl groups in the phenyl scaffold could be responsible for both the disruption and formation of ligand-protein interactions with a final decreased effect in the experimental activity. Moreover, the position of the third hydroxyl group is close to some hydrophobic residues, such as Leu286 and Val288 that define the acyl-binding pocket in the BChE. Polar substituents near this region could contribute to reduce the energy binding.

**Figure 7:**
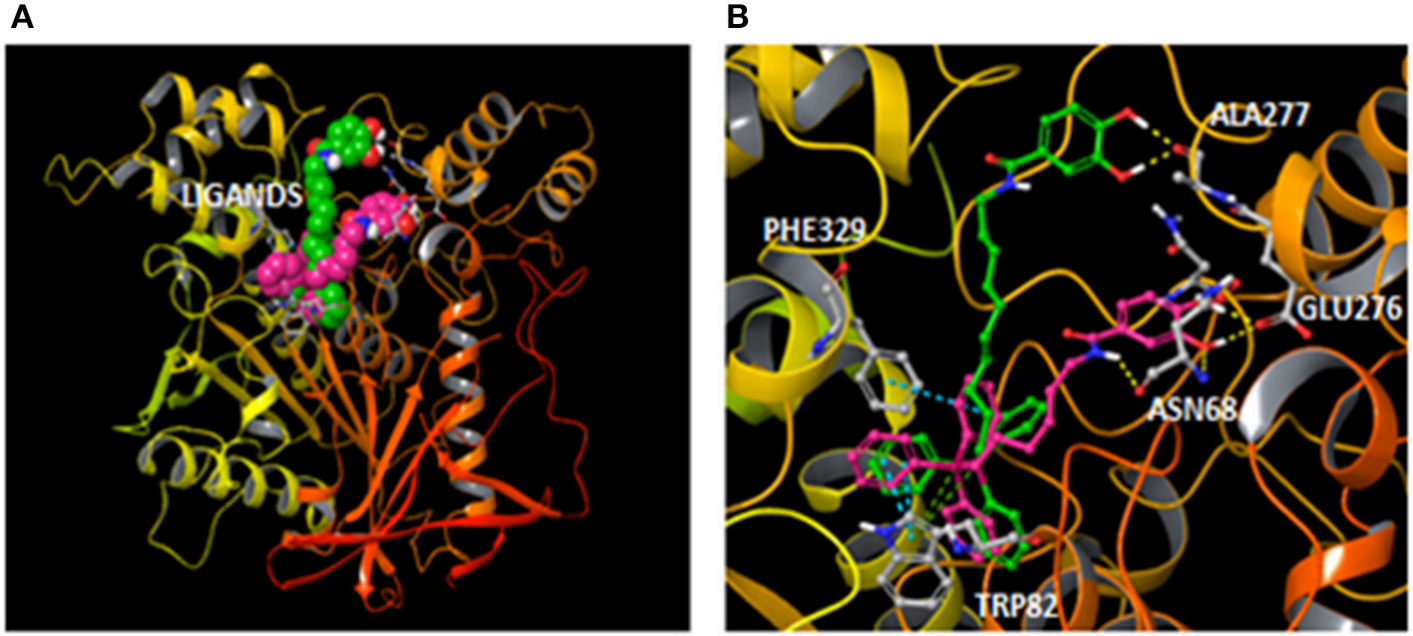
**(A)** General overview with compounds **AntiOxBEN**_**1**_ and **17** (pink and green carbons respectively) bound to BChE after docking simulations; and **(B)** Comparison of the hypothetical binding modes determined for compounds **AntiOxBEN**_**1**_ (pink carbons) and **17** (green carbons) inside the BChE (color code: yellow dashes for hydrogen bonds, blue dashes for π-π stacking interactions and green dashes for π-cation interactions).

On the other hand, the enlargement of the aliphatic chain caused a slight reduction of the BChE activity. Molecular docking showed a binding mode for compound **17** with some differences regarding **AntiOxBEN**_**1**_. Since compound **17** has an extended spacer length, the protein pocket accommodated the compound in a different binding position that could be responsible for the reduction of the activity. The hydroxybenzamide group was accommodated in a shallower region of the protein pocket and established hydrogen bonds with the residue Ala277 (Figure 7). However, the different position of the hydroxybenzamide scaffold did not yield hydrogen bonds with residues Asn68 and Glu276 as **AntiOxBEN**_**1**_. The proposed different binding mode for compound **17**, due to the enlargement of the aliphatic chain between the triphenylphosphonium and the hydroxybenzamide groups, could be responsible for the reduction of BChE activity.

According to molecular modeling studies, this type of mitochondriotropic antioxidants are able to interact with both catalytic active site (CAS) and PAS of BChE and so they can act as bifunctional inhibitors (Brunhofer et al., 2012; Eckroat et al., 2013).

## Conclusion

For the first time, dual target compounds based on mitochondriotropic antioxidants endowed with cholinesterase bifunctional inhibitory activity were described.

**AntiOxBEN**_**1**_ acted as a selective and non-competitive BChE inhibitor and compounds **15–18** showed similar antioxidant and ChE inhibitory activities, when compared to **AntiOxBEN**_**1**_ and **AntiOxBEN**_**2**_. From the series, **AntiOxBEN**_**1**_ and compound **18** were the most promising BChE and/or AChE inhibitors, respectively, acting by a non-competitive mechanism. Moreover, these compounds did not exhibit cytotoxic effects in both SH-SY5Y and HepG2 cells and significantly prevented Aβ1–42 peptide-induced dysfunction/cell death. Dual inhibition may also help to slow down the formation of amyloidogenic compounds, providing an important neuroprotective disease-modifying effect.

According to the BChE selectivity and antioxidant properties as well as drug-like properties, which points toward BBB permeability, and favorable toxicological profile the mitochondriotropic antioxidant **AntiOxBEN**_**1**_ is considered a valid candidate for the development of a dual acting drugs useful for AD therapy, which should be validated in animal models relevant for the disease.

## Author contributions

CO: Synthesized the compounds, performed biological experiments, analyzed the data and wrote the paper; FC and LS: Helped in synthesis; JT and RA: Performed biological experiments, analyzed the data and refined the manuscript; FM, TS, JG, and FR: Helped in data analysis and also refined the manuscript; SV: Performed the molecular docking studies; EU, PO, and FB: Designed the project, experiments, and wrote the paper. All authors revised the manuscript and agreed with submission.

### Conflict of interest statement

PO and FB are co-founders of the start-up company MitoTAG. The other authors declare that the research was conducted in the absence of any commercial or financial relationships that could be construed as a potential conflict of interest.

The handling Editor declared a shared affiliation, though no other collaboration, with one of the authors, FM.

## Abbreviations

ABTS^•+^: 2,2′-azino-bis(3-ethylbenzthiazoline-6-sulfonic acid)
ACh: Acetylcholine
AChE: acetylcholinesterase
AD: Alzheimer's disease
ATCI: Acetylthiocholine iodide
BBB: blood-brain barrier
BCh: butyrylcholine
BChE: butyrylcholinesterase
BTCI: butyrylthiocholine iodide
ChEs: cholinesterases
clogP: logarithm of the octanol-water partition coefficient
CNS: central nervous system
DIPEA: *N,N*-diisopropylethylamine
Diphos: 1,2-bis(diphenylphosphine)ethane
DPPH^•^: 2,2′-diphenyl-1-picrylhydrazyl radical
DTNB: 5,5′-dithiobis-(2-nitrobenzoate)
EDTA: ethylenediaminetetraacetic acid
FDA: Food and Drug Administration
GA: gallic acid
HBA: number of hydrogen acceptors
HBAc: hydroxybenzoic acid(s)
HBD: number of hydrogen donors
HBSS: Hank's balanced salt solution
*Ki*: inhibition constant
*K*_*m*_: Michaelis constant
log BB: blood (plasma)-brain partitioning
NEAA: nonessential amino acids
*n*rotb: number of rotatable bonds
OS: oxidative stress
PA: protocatechuic acid
PPh_3_: triphenylphosphine
ROS: reactive oxygen species
TNB^2−^: anion 5-thio-2-nitrobenzoate
TPA: 12-*O*-Tetradecanoylphorbol-13-acetate
TPP^+^: triphenylphosphonium cation
tPSA: topological polar surface area
*V*_*max*_: maximum velocity
ΔΨ: transmembrane electric potential.
